# Malaria resistance-related biological adaptation and complex evolutionary footprints inferred from one integrative Tai-Kadai-related genomic resource

**DOI:** 10.1016/j.heliyon.2024.e29235

**Published:** 2024-04-09

**Authors:** Shuhan Duan, Mengge Wang, Zhiyong Wang, Yan Liu, Xiucheng Jiang, Haoran Su, Yan Cai, Qiuxia Sun, Yuntao Sun, Xiangping Li, Jing Chen, Yijiu Zhang, Jiangwei Yan, Shengjie Nie, Liping Hu, Renkuan Tang, Libing Yun, Chuan-Chao Wang, Chao Liu, Junbao Yang, Guanglin He

**Affiliations:** a Institute of Basic Medicine and Forensic Medicine, North Sichuan Medical College and Center for Genetics and Prenatal Diagnosis, Affiliated Hospital of North Sichuan Medical College, Nanchong, Sichuan, 637007, China; b Institute of Rare Diseases, West China Hospital of Sichuan University, Sichuan University, Chengdu, 610000, China; c Research Center for Genomic Medicine, North Sichuan Medical College, Nanchong, 637100, China; d Department of Forensic Medicine, College of Basic Medicine, Chongqing Medical University, Chongqing, 400331, China; e School of Forensic Medicine, Shanxi Medical University, Jinzhong, 030001, China; f School of Forensic Medicine, Kunming Medical University, Kunming, 650500, China; g West China School of Basic Science & Forensic Medicine, Sichuan University, Chengdu, 610041, China; h State Key Laboratory of Cellular Stress Biology, National Institute for Data Science in Health and Medicine, School of Life Sciences, Xiamen University, Xiamen, 361005, Fujian, China; i Anti-Drug Technology Center of Guangdong Province, Guangzhou, 510230, China; j Center for Archaeological Science, Sichuan University, Chengdu, 610000, China

**Keywords:** Biological adaptation, Genetic admixture, Population history, Tai-Kadai people, Malaria resistance

## Abstract

Pathogen‒host adaptative interactions and complex population demographical processes, including admixture, drift, and Darwen selection, have considerably shaped the Neolithic-to-Modern Western Eurasian population structure and genetic susceptibility to modern human diseases. However, the genetic footprints of evolutionary events in East Asia remain unknown due to the underrepresentation of genomic diversity and the design of large-scale population studies. We reported one aggregated database of genome-wide SNP variations from 796 Tai-Kadai (TK) genomes, including that of Bouyei first reported here, to explore the genetic history, population structure, and biological adaptative features of TK people from southern China and Southeast Asia. We found geography-related population substructure among TK people using the state-of-the-art population genetic structure reconstruction techniques based on the allele frequency spectrum and haplotype-resolved phased fragments. We found that the northern TK people from Guizhou harbored one TK-dominant ancestry maximized in the Bouyei people, and the southern TK people from Thailand were more influenced by Southeast Asians and indigenous people. We reconstructed fitted admixture models and demographic graphs, which showed that TK people received gene flow from ancient southern rice farmer-related lineages related to the Hmong-Mien and Austroasiatic people and from northern millet farmers associated with the Sino-Tibetan people. Biological adaptation focused on our identified unique TK lineages related to Bouyei, which showed many adaptive signatures conferring Malaria resistance and low-rate lipid metabolism. Further gene enrichment, the allele frequency distribution of derived alleles, and their correlation with the incidence of Malaria further confirmed that *CR1* played an essential role in the resistance of Malaria in the ancient "Baiyue" tribes.

## Abbreviations

TK: Tai-Kadai
TB: Tibeto-Burman
HM: Hmong-Mien
AN: Austronesian
AA: Austroasiatic
AR: Amur River
YRB: Yellow River Basin
MSEA: Mainland Southeast Asian
SEA: Southeast Asian
YBP: Years before the present
BBQC: BaBanQinCen
WGS: Whole-genome sequencing
mtDNA: Mitochondrial DNA
STRs: Short Tandem Repeats
SNP: Single Nucleotide Polymorphism
BBN: Bouyei samples from Guizhou Bannong
HGDP: Human Genome Diversity Project
HO: Human Origin
F_ST_: Fixation index
PCA: Principal component analysis
LD: Linkage disequilibrium
ROH: Runs of homozygosity
IBD: Identity by Descent
Ne: Effective population size
ALDER: Admixture-induced Linkage Disequilibrium for Evolutionary Relationships
PBS: Population Branch Statistic
LA: Linoleic acid
FADS: Fatty acid desaturase
EDAR: Ectodysplasin A receptor
PUFA: Polyunsaturated fatty acid
HLA-DPA1: Major Histocompatibility Complex, Class II, DP Alpha 1
CR1: Complement C3b/C4b Receptor 1, Knops Blood Group
RBCs: Red blood cells
CLSTN2: Calsyntenin 2
TMEM258: Transmembrane Protein 258
TMEM121: Transmembrane Protein 121
FEN1: Flap Structure-Specific Endonuclease 1
MYRF: Myelin Regulatory Factor
PTPRD: Protein Tyrosine Phosphatase Receptor Type D
10K_CPGDP: 10K Chinese People Genomic Diversity Project
AADR: Allen Ancient DNA Resource

## 1 Introduction

East Asia is one of the densest population residence areas in the world and is characterized by abundant cultural, linguistic, and genetic diversity. Ancient East Asian genomes revealed a high degree of genetic differentiation and large-scale population admixture between ancient northern and southern East Asians since the early Neolithic period [1]. It was common to observe genetic stability or continuity in the Tibetan Plateau, Amur River Basin (AR), Fujian, and Taiwan Island, which differed from the massive migrations and complex admixture scenarios observed in Europe and Southeast Asian (SEA) [[2], [3], [4], [5]]. Agriculture-derived population expansion and migration shaped the genetic and linguistic diversity patterns in the core regions of China and SEA, supporting the farming-language-people dispersal hypothesis [5,6]. People spread around the AR in northern China, mainly accompanied by the spread of the Altaic languages [3]. Millet farmers dispersed from the Yellow River Basin (YRB) in Central China, and this population expansion was associated with the spread of Tibeto-Burman languages across eastern Eurasia [7]. Rice farmers migrated from the Yangtze River Basin accompanied by the spread of Hmong-Mien (HM), Tai-Kadai (TK), Austroasiatic (AA), and Austronesian (AN) languages in South China [3].

Southern China is the birthplace of rice-cultivating agriculture and is a pivotal crossroad for rice farmers migrating southward to SEA [8]. Moreover, abundant linguistic, cultural, and ethnic diversity contributed to the mysterious verve of the evolutionary history of southern China [[9], [10], [11]]. The TK-speaking populations were the indigenous people of southern China and were widely distributed in southern China, mainland Southeast Asian (MSEA), and southern Asia, ranging from Hainan Island in the east to northeast India in the west and from southern Sichuan in the north to southern Thailand in the south. According to archaeological and historical documents, the ancient "Baiyue" living in Southeast China was considered the ancestor of present-day TK-speaking populations [12]. During the Han Dynasty, under the pressure of war and famine, numerous "Baiyue" people expanded southward to Southwest China and SEA for long periods. Ancient DNA evidence has shown that the Bronze Age migration of farming people brought TK ancestry and culture to SEA [5,6]. Innumerable subsequent isolation and genetic admixture events further shaped the specific patterns of the genetic structure of present-day TK-speaking populations [13]. However, due to the geographical proximity of the TK people to other southern Chinese groups (HM, AN, and AA), the genetic origins of TK people, phylogenetic relationships between TK-speaking populations and geographical neighbors, and genetic signatures of pathogen‒host interactions remain to be fully characterized.

Previous work provided new insights into the population history of the Proto-TK people and their interactions with modern and ancient Southeast Chinese people [14,15]. In addition, genetic evidence based on the Y chromosome, mitochondrial DNA (mtDNA), and forensic-related low-resolution genetic variations have provided essential clues for identifying geographically restricted TK patients in China [14,15]. Cultural documents suggest that hanging coffin relics in southern China and SEA share many common cultural elements with ancient "Baiyue" tribe relics, further providing cultural evidence for the South China origin of the TK people. Zhang et al. reported that ancient southern Chinese populations approximately 3600 years before the present (YBP) associated with hanging coffins originated from the coastal region of southern China (likely the Mount Wuyi region of China) based on maternal genetic evidence [16]. Wang et al. identified two historic populations in Guangxi that were strongly associated with modern linguistically different people: 1500-year-old BaBanQinCen (BBQC), which is related to TK speakers, and 500-year-old Gaohuahua, which is connected to HM speakers [17]. Contemporary genetic evidence has also provided new insights into the population formation of TK people. He et al. found an excellent representative source for TK people on Hainan Island [15]. Chen et al. investigated the admixture history of the Hainan Li people based on whole-genome sequencing (WGS) data and revealed that TK-speaking populations from South China and North Vietnam showed close genetic affinity, which suggested a common genetic origin of geographically different "Baiyue" lineages [18]. In addition, this work also estimated the formation time of the Li-specific lineage O-M95 and refined the possible divergence time of the "Baiyue" lineage approximately ∼11,000 years ago [18].

Nevertheless, the lack of systematic research on the genetic substructure of TK people in inland China has hindered our understanding of the whole landscape of TK speakers. Preceding genetic analyses were mainly focused on single inland TK-speaking populations or geographically restricted groups based on low-density forensic genetic markers (such as traditional Y and mtDNA genetic markers) or overlapping low-density 50K SNPs [[19], [20], [21], [22], [23], [24]]. Therefore, intensive and in-depth genetic studies focused on the evolutionary features of TK people are needed. More efforts should be made to focus on multiregional integration, systematic scale descriptions, complex population modeling, and biological adaptation, especially for the mountain population of Bouyei. The Bouyei are an ethnic group that mostly lives in Guizhou Province [25]. Guizhou has complex landforms and numerous mountain ranges. It is an essential part of the Yungui Plateau and is geographically close to Yunnan and Guangxi provinces, which possess substantial sociocultural, genetic, and linguistic diversities [26]. There are more than 20 officially recognized or unrecognized ethnic groups widely distributed in Guizhou, and the complexity of geographical environments and ecological diversities further provide favorable conditions for forming the unique genetic structure of these ethnic groups. Bouyei is among the 18 officially recognized ethnic minorities in Guizhou Province and is mainly distributed in Yunnan, Sichuan, and other provinces. Among these, Guizhou has the largest population of Bouyei, accounting for approximately 97 % of the Bouyei population in China. The national language of Bouyei belongs to the TK (also known as Kra-Dai) language family, and its ancient ancestors have inhabited Guizhou Province since the Stone Age and grew rice and other crops for a living. Ren et al. performed a preliminary exploration focused on the genetic structure of Bouyei populations in Guizhou based on short tandem repeats (STRs) on X chromosomes [27]. He et al. also explored the genetic diversity and forensic characteristics of Bouyei based on insertion/deletion markers [14].

Previous genetic studies on the forensic characteristics and population admixture of the Bouyei Group were primarily based on low-density genetic markers, while the fine-scale genetic structure and detailed genetic history of the Bouyei Group remain unclear. Thus, we performed a comprehensive population genetic analysis to describe their ancestral composition and reveal their genetic origin. This contributed to exploring the population structure on a fine scale, reconstructing the evolutionary history of inland TK-speaking populations, and enriching the available genomic resources. To systematically explore the genetic structure, reconstruct demographic events, and resolve the environmental adaptation of geographically different TK-speaking populations, we collected new Bouyei samples from Guizhou Bannong (BBN) and merged our data with publicly available array-based genotyping data from modern and ancient Eurasian populations. In this study, we aimed to answer the following four questions: (1) What are the overall patterns of genetic diversity in TK people, and what are the impacts of geographical and cultural factors on genetic diversity? (2) How many ancestral sources contributed to the gene pool of modern TK people, and what role does Bouyei-related ancestry play in this process? (3) How do genetic homogeneity and heterozygosity differ among ethnically different TK people from South China and SEA and geographically different Bouyei people inferred from fine-scale shared haplotypes and allele frequency spectrum? (4) In the annals of history and epidemiological data, southern China has emerged as a focal point for increased malaria incidence. Furthermore, natural selection signals related to malaria have also been distinguished in Li populations in Hainan [18]. However, whether and to what extent inland TK people have adapted to the environment exposed to malaria remains unknown. What are the influences of selection pressures on the genetic architecture of TK people, and what is the genetic legacy of their interaction with ancient Malaria-related infectious diseases? We provided new insights into the genetic admixture history of TK people (especially newly reported Bouyei-related ancestry) and inferred signatures of natural selection in mountainous circumstances based on 796 TK genomes.

## 2 Results

### 2.1 General patterns of population structure of TK people in the context of worldwide populations

**Fig. 1:**
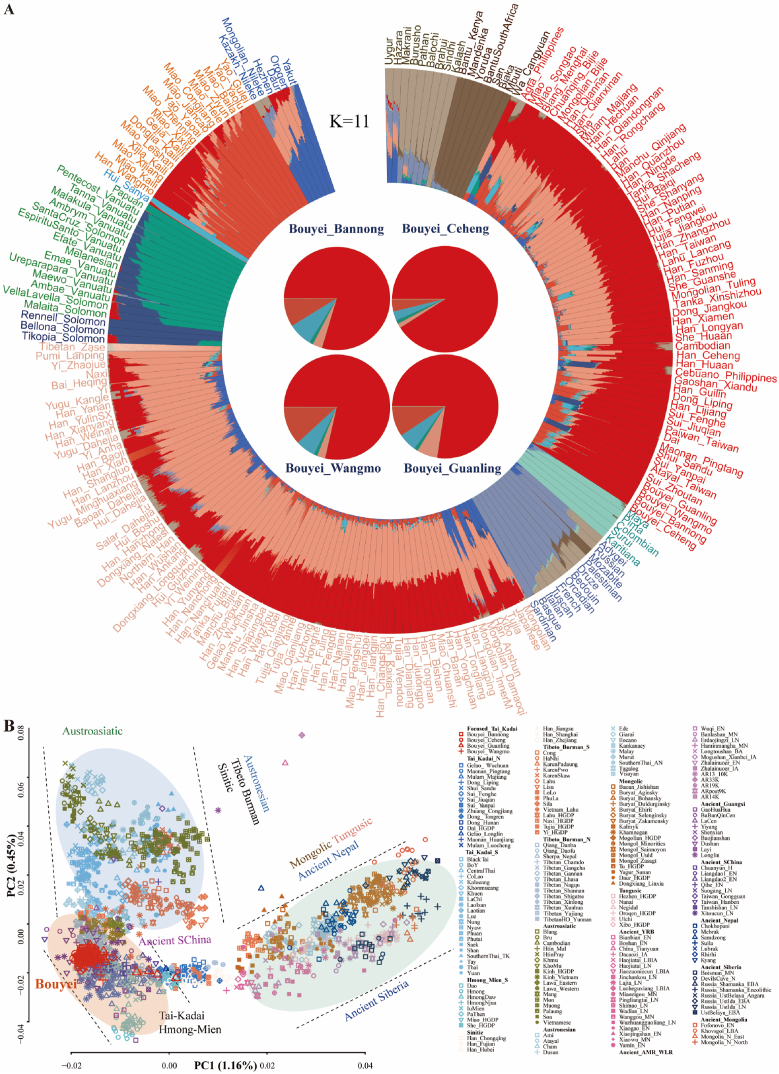
General genetic affinity and population structure among different modern and ancient populations. (**A**) Results of model-based ADMIXTURE clustering analysis. Based on a high-density dataset, the clustering patterns were visualized for the 207 populations at K = 11. Different colors represent different ancestral components. (**B**) PCA results showing the patterns of genetic relationships based on a low-density merged HO dataset. An East Asian-based PCA was conducted based on the genetic variations of modern and ancient people. Ancient people were projected onto it. (For interpretation of the references to color in this figure legend, the reader is referred to the Web version of this article.)

To dissect the ancestral components and genetic similarity of the TK speakers, we conducted a model-based ADMIXTURE analysis among 207 modern worldwide populations from our previously published data and reference data from the Human Genome Diversity Project (HGDP) and Oceanian genomic resources [28,29]. When K = 11, we observed five East Asian-related ancestries [Yakut-related (blue), Yao-related (orange), Hui-related (light blue), Tibetan-related (light orange), and Bouyei-related (red)] and six non-East Asian ancestries [Papuan-related (dark green), Solomen-related (dark blue), Sardinian-related (light purple), Karitiana/Surui-related (light green), Mbuti-related (brown), and Kalash-related (light brown)] (Fig. 1A and S1). The red ancestry component widely existed in our studied Bouyei and their neighbors and was first reported as inland TK ancestry in our work. Bouyei had the highest proportion of this newly identified ancestry (79.5 % ± 0.0885), followed by Yao (9 %) and Hui (7.5 %) from South China, and Tibetans had less inland TK-related ancestry. However, this could not be ignored, which suggested that inland TK-related ancestry played an essential role in the formation of ancient Bouyei and modern southern Chinese people. Other TK people had similar admixture patterns with different proportions of inland TK-related ancestry (Fig. S2). Notably, the ancestral proportions of northern East Asians (Tibetan-related component) in some TK-speaking populations were remarkable, especially in Gelao, which was markedly different from the ancestral composition patterns observed in our studied Bouyei groups (Fig. S2).

To explore the genetic affinity between Chinese TK people and southern TK people from Vietnam, Thailand, and Laos (Fig. 1B), we merged our genome-wide data with publicly available 1991 modern and ancient individuals from 207 populations genotyped via Affymetrix Human Origin array and formed the second dataset, which was referred to as the low-density merged HO dataset and included 56,814 SNPs (Table S1, Fig. 1B). We conducted principal component analysis (PCA) with ancient people projected onto modern people's genetic backgrounds and identified three clines related to the northern Altaic cline, central HM/TK cline, and southern AA/AN/TK-related cline. The first component, which explained 0.71 % of the variance, distinguished HM and TK speakers from Tungusic and Mongolic populations in North China and ancient Siberians. The second component (PC2: 0.45 %) separated HM/TK speakers from AN/AA-speaking people. The ancient people from Guangxi, including the historic Gaohuahua and BBQC, were closely clustered with the Chinese HM and TK people, respectively.

**Fig. 2:**
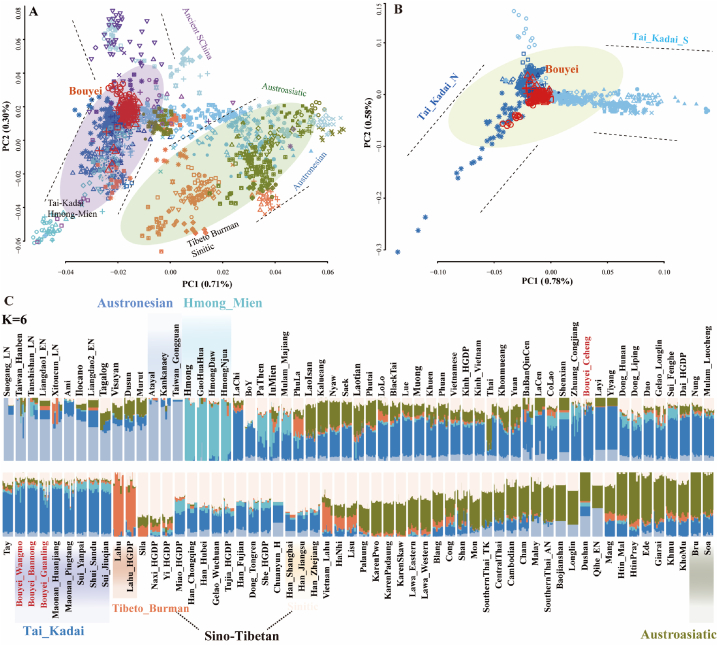
Apparent genetic substructure among 39 TK-speaking populations and other East Asian reference populations. (**A**) PCA results showing the genetic relationships among 117 South Chinese populations. Analysis was conducted based on the genetic variations of modern and ancient people, where ancient people were projected onto it. The colors used here are consistent with those in Fig. 1B, which represent linguistically different modern populations and spatiotemporally different ancient populations. (**B**) PCA among 39 TK-speaking populations. (**C**) Model-based ADMIXTURE results showing population clustering patterns of both modern and ancient populations. We used six predefined ancestral sources as the lowest cross-validation error value. (For interpretation of the references to color in this figure legend, the reader is referred to the Web version of this article.)

Furthermore, we explored the admixture history of TK-speaking populations in the context of modern and ancient southern East Asia. We observed that TK-speaking populations were mainly HM-speaking populations and were far from AA/AN/TB-speaking populations; they also showed close genetic similarity with ancient people from Guangxi, Fujian, and surrounding regions, which implied that the ancestors of TK-speaking populations were possibly relevant to the descendants of southern Chinese individuals (Fig. 2A). Moreover, we focused on the demographic events that occurred within all 39 TK-speaking populations from MSEA and southern China to explore the fine-scale genetic affinities (Table S2, Fig. S3). PCAs based on linkage disequilibrium (LD)-independent SNPs showed that the dispersion of TK-speaking populations was related to their geographical locations, and both longitude and latitude were negatively correlated with PC1 values (Fig. 2B and S4). The ADMIXTURE results of 117 southern Chinese populations with K = 6 showed that the TK-related component gradually decreased from north to south, and TK-speaking populations from MSEA derived more ancestral components from AA-related ancestry than did TK-speaking populations from China (Fig. 2C).

### 2.2 Genetic substructure among 39 TK-speaking populations

**Fig. 3:**
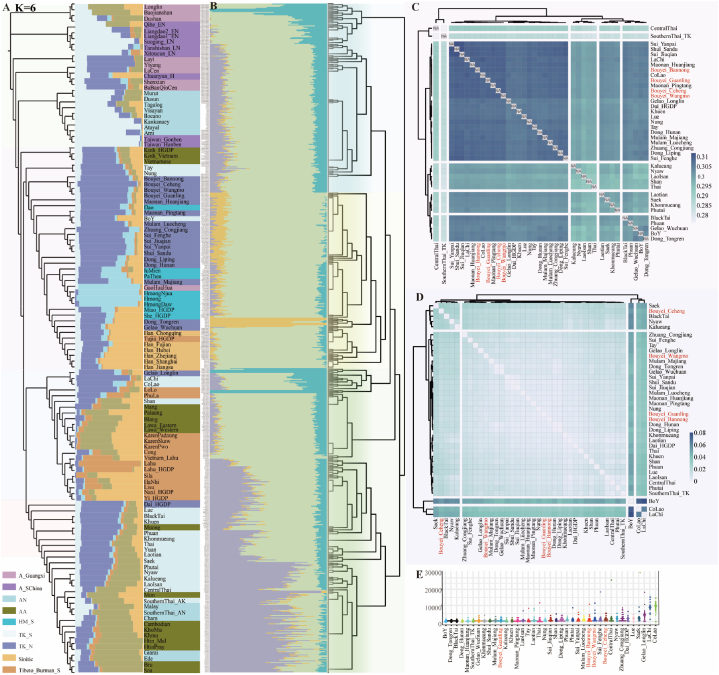
Clustering patterns of 39 TK-speaking populations and East Asian reference populations and their neighbors. (**A**) A phylogenetic tree reconstructed based on the matrix of 1-outgroup *f*_3_ showing the topological pattern of 117 populations from southern China and SEA (left). The bar plot shows the mean ADMIXTURE-based admixture proportions with six predefined ancestral sources. The lowest cross-validation error value was observed when six ancestral sources were used among the 117 modern and ancient populations (right). (**B**) Fine-scale genetic structure among 796 individuals from 39 TK-speaking populations inferred from their ADMIXTURE-fitted model with four predefined ancestral sources (left) and a fine-STRUCTURE-based dendrogram based on the coancestry coefficient (right). (**C**) Heatmap of the shared genetic drift inferred from outgroup *f*_3_-statistics in the form *f*_3_(Studied Bouyei, TK; Mbuti) among 39 populations from southern China and SEA. (**D**) Heatmap of pairwise Fst genetic distances among 39 TK-speaking populations. (**E**) The mean lengths of runs of homozygosity for 39 TK-speaking populations.

The patterns of genetic affinity of TK-speaking populations inferred from PCA and model-based ADMIXTURE significantly differed from those observed in geographically distinct TB people from the Tibetan Plateau and other geographical edge populations. Most TK people had multiple ancestral components and were clustered in an intermediate position in the East Asian-scale PCA. To further illuminate the genetic similarities and differences within geographically different TK-speaking populations and explore their fine-scale population substructure, we conducted population genetic analysis and admixture modeling within 796 individuals from 39 TK-speaking populations based on the merged low-density HO dataset, which included 56,814 SNPs. The genetic patterns revealed by the ADMIXTURE results were consistent with those observed in the PCA, which indicated that geographically different TK people possessed varying proportions of ancestral components and different genetic admixture histories. The predefined ancestral sources increased from 6 to 10, which generally led to a single ancestral population component being distinguished when K = 6 had the lowest cross-validation error, suggesting their extensive genetic substructure (Fig. S5). To further investigate the genetic differentiation of the TK-speaking populations, we constructed a phylogenetic tree based on 1-outgroup-*f*_*3*_ among 117 southern Chinese populations (Table S3, Fig. 3A). We found that ancient people from South China, except for Gaohuahua, clustered more closely with modern AN speakers than with other reference populations, and the 500-year-old Gaohuahua people clustered with HM-speaking people. We observed three main branches among modern populations: the upper branch included northern TK, northern TB, HM, and their geographical neighbors, and people from similar language families or geographical locations gathered with each other; the middle branch included southern TB and their neighbors; and the lower branch included southern mainland TK and AN people. The Bouyei groups generally clustered with geographically close TK-speaking populations, such as the Maonan, Zhuang, and Shui populations from Guizhou Province.

We further explored genetic differentiation and substructure using haplotype-based fine structure and admixture model reconstructions based on the allele frequency spectrum among 796 individuals (Fig. 3B). Haplotype-based clustering patterns were consistent with the model-based ADMIXTURE ancestral composition, in which most TK individuals possessed primary simulated ancestry maximized in northern Guizhou and southern Thailand people. The pattern of genetic similarity inferred from the 1-outgroup-*f*_3_ heatmaps also revealed differentiated patterns of shared alleles between the northern and southern TK-speaking populations (Fig. 3C and S6). We observed a strong genetic affinity between the Bouyei population and geographically proximate Chinese TK-speaking populations, consistent with the identified genetic relationships based on pairwise F_ST_ genetic distance (Fig. 3D–Table S4). Moreover, we investigated the correlations between the length and number of runs of homozygosity (ROH) across various ethnic minorities and further explored the distribution pattern (Fig. 3E). The total lengths of ROH in TK-speaking populations from the MSEA were relatively greater, whereas those in Chinese TK-speaking populations exhibited a similar distribution pattern. Generally, we highlighted geography-related genetic substructure within 39 TK-speaking populations, including southern TK-speaking populations in the MSEA and northern TK-speaking populations in southern China.

**Fig. 4:**
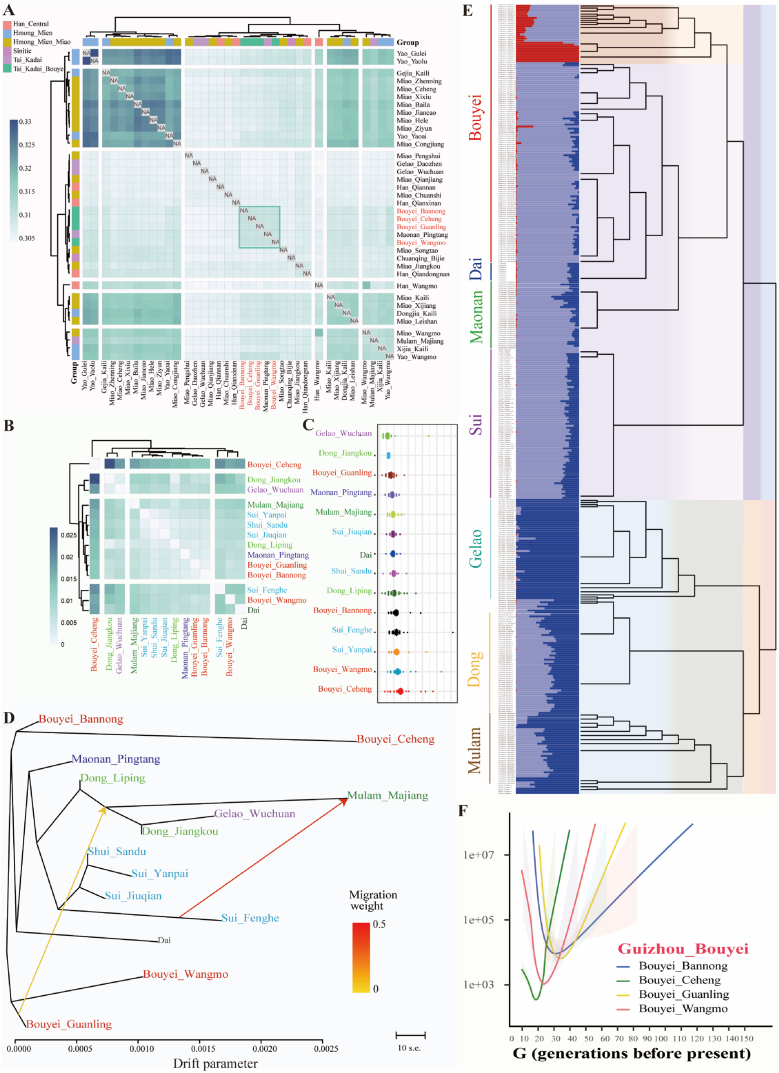
The reconstructed demographic history model based on the high-density genomic source showed that four Bouyei populations have a close genetic relationship with surrounding populations in Guizhou. (**A**) A heatmap of the shared genetic drift inferred from outgroup *f*_3_-statistics in the form of *f*_3_(Studied Bouyei, Reference; Mbuti). (**B**) Heatmap of pairwise Fst genetic distances among 14 TK-speaking populations in Guizhou Province. (**C**) The mean lengths of runs of homozygosity within 14 TK-speaking populations. (**D**) The phylogenetic relationship showed the close genetic affinity between Bouyei and other TK-speaking Guizhou populations. (**E**) The combined results from the best-fit ADMIXTURE with three ancestral sources (K = 3, left) and the fineSTRUCTURE-based dendrogram (right)(**F**) The effective population size of four Bouyei populations from 150 generations before the present.

Population genetic structure analyses in the context of East Asians and regional TK people identified one unique ancestry dominant in Guizhou TK people. The complexity of geographical environments and unique cultures indirectly provide favorable conditions for the formation of various ethnic groups and genetically differentiated population structures [25]. To determine the confidence of the newly identified ancestry component and explore its interaction with geographically different Guizhou people, we performed a population comparison analysis among Guizhou populations, including HM/TB/Sinitic and TK-related populations. We observed that Bouyei shared the most alleles with the surrounding TK-speaking populations and other geographically proximal ethnic minorities (Fig. 4A). Shared alleles and haplotypes further confirmed the high genetic affinity among geographically different TK-speaking populations and that these populations frequently interacted with each other (Fig. 4B and S7). The ROH values of the Bouyei populations were relatively high, and the distribution patterns showed remarkable similarities to those of neighboring ethnic minorities, such as Dong and Shui (Fig. 4C), which indicated the possibility of inbreeding [30]. We also reconstructed the maximum-likelihood-based TreeMix among 14 TK-speaking populations to investigate phylogenetic relationships (Fig. 4D and S8). TreeMix-based phylogenetic relationships demonstrated the population substructure among TK-speaking populations and indicated that BBN had close genetic affinities with other geographically different Bouyei groups when two gene flow events occurred. To determine the effective population size (N_e_) within the past 150 generations, we identified bottleneck events at different times and evaluated genetic diversity among TK-speaking populations (Fig. 4F). Additionally, we explored the genetic differentiation and patterns of fine-scale genetic structure using fineSTRUCTURE (Fig. 4E), which was consistent with the patterns of population substructure revealed by the pairwise coincidence matrix (Fig. S9).

### 2.3 The shared alleles revealed by *f*-statistics

**Fig. 5:**
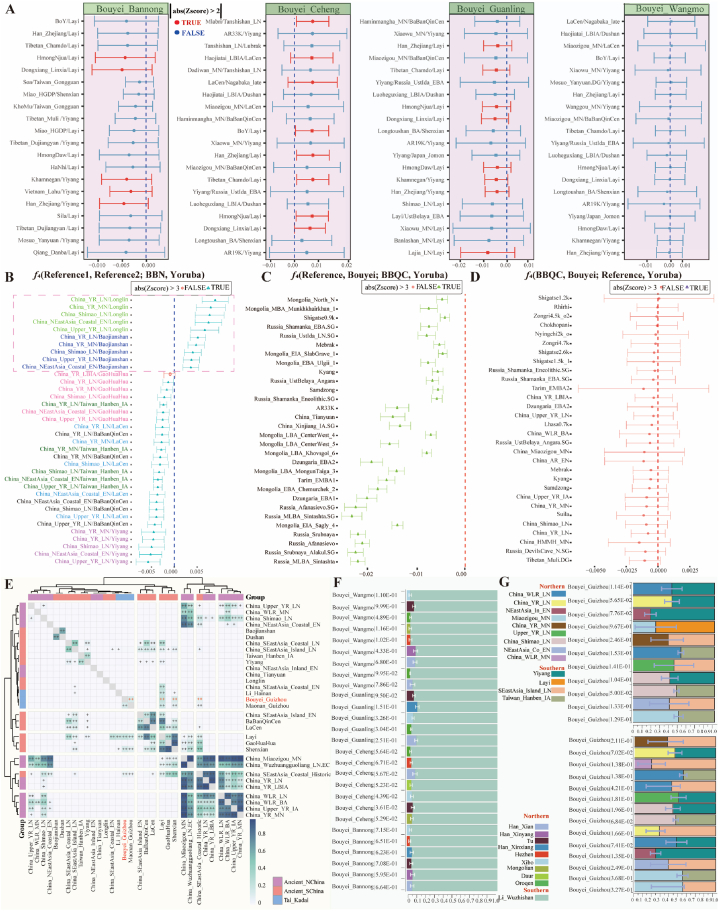
**The estimated admixture signatures, differentiated shared alleles, and admixture models of Bouyei speakers**. (**A**) Admixture-*f*_3_-statistics in the form *f*_3_(Modern/Ancient Reference 1, Modern/Ancient Reference 2; Bouyei). A Z-score lower than −2 was used to highlight possible admixture signals. We presented the top 20 source pairs for each targeted population. Red indicates that the Z-values are significant, and blue indicates that the Z-values are not statistically significant. (**B**) *F*_4_-statistics test in the form of *f*_4_(Reference population1, Reference population2; BBN, Mbuti) based on the middle-density merged 1240K dataset identified the genetic affinity between BBN and other references. BBN: Bouyei_Bannong, BBQC: BaBanQinCen. (**C**) *F*_4_-statistics in the form of *f*_4_(Reference, *Meta*-Bouyei; BBQC, Mbuti) were used to explore the genetic associations within *Meta*-Bouyei and BBQC. (**D**) *F*_4_-statistics in the form of *f*_4_(BBQC, *Meta*-Bouyei; Reference, Mbuti) were used to verify whether references except BBQC influenced *Meta*-Bouyei. (**E**) Pairwise qpWave results showing the genetic homogeneity and heterogeneity between *Meta*-Bouyei and other ancient populations. The label "+" denotes p values greater than 0.01. (**F**) Two-way admixture models showing that both the modern northern and southern populations contributed to the formation of the four Bouyei people. The error bars indicate the standard errors of the predicted proportions of ancestors obtained from qpAdm. (**G**) Two-way admixture models showing that ancient northern and southern populations contributed to the formation of the *Meta*-Bouyei people. The error bars indicate the standard errors of the predicted proportions of ancestors obtained from qpAdm. (For interpretation of the references to color in this figure legend, the reader is referred to the Web version of this article.)

To explore the detailed demographic history and possible ancestral sources of the Bouyei populations, we conducted admixture *f*_3_-statistics in *f*_3_(Reference population1, Reference population2; Studied populations) based on the HO dataset (Fig. 5A). We did not observe significant *f*_3_ values in the Bouyei groups. We cannot wholly exclude ancient or recent admixture events with subsequent substantial population bottlenecks. We further performed ChromoPainterv2 to determine the ancestral haplotype composition and ran a fastGLOBETROTTER based on haplotype sharing to identify ancestral sources and date and describe admixture events of our targeted populations. For the BBN groups, the best-guess conclusion for admixture was "unclear signal", which provided evidence about the unique population demographic history and suggested a relatively isolated genetic background. We then performed a series of *f*_4_-statistics to explore genetic differentiation and gene flow events between the Bouyei population and other populations. Interestingly, we did not observe significant negative signals except for spurious signals (Fig. S10). Moreover, we observed the same result based on the genetic variations of the modern populations (Table S5), which confirmed the relative genetic homogeneity within the four Bouyei groups.

To further explore the genetic affinity between BBN and the reference populations, we conducted *f*_4_-statistics in the form of *f*_4_(Reference population1, Reference population2; BBN, Mbuti). When focused on ancient populations, we found that the studied groups were affected by northern and southern gene flow. BBN shared more alleles with ancient northern East Asian populations than with early Neolithic southern populations (Baojianshan/Longlin). BBN received more genetic influence from ancestry related to the Guangxi historical people (BBQC), earlier Fujian Neolithic individuals, and Taiwan Island Hanben people compared with ancient YRB farmers (Fig. 5B). For modern East Asian populations, BBN had a closer genetic relationship with southern populations than with northern Chinese populations (Tables S6∼9). Within southern Chinese populations, we observed that Bouyei populations shared more alleles with HM in *f*_4_(AN, HM; BBN, Mbuti). Consistent with the genetic relationship inferred from outgroup *f*_3_ (Fig. 4A), the Bouyei populations had a closer genetic affinity with other geographically close TK-speaking populations in *f*_4_(TK, HM; BBN, Mbuti). In addition, to further explore the general ancestral sources of the Bouyei populations and improve their statistical power, we merged four Bouyei groups into a *meta*-Bouyei population, as their genetic homogeneity was identified via pairwise qpWave analysis (Figures S10 and 11). According to archaeological evidence [17], the BBQC was a historical population living in Guangxi Province 1500 years ago and is considered the direct ancestor of the TK people. We then performed *f*_4_-statistics in the form of *f*_4_(Reference populations, *Meta*-Bouyei; BBQC, Mbuti) and found abundant significant negative *f*_4_ values, which suggested that our studied groups shared more alleles with BBQC than other references (Fig. 5C). Additionally, the Bouyei populations were influenced by gene flows from Tibetan-related ancestral sources (Muli Tibetan: Z = −2.572) in the form of *f*_4_(BBQC, *Meta*-Bouyei; Reference populations, Mbuti) (Fig. 5D), which was consistent with the heterogeneity observed within the Bouyei populations and BBQC (Fig. 5E). Overall, different types of *f*_4_ analyses revealed strong genetic affinity within TK-speaking populations and demonstrated a common origin in the Bouyei population.

We built two-way qpAdm admixture models with Han and other Altaic people as the northern Chinese surrogates and the island Li people as the southern surrogates to directly explore the admixture sources and proportions of our newly studied Bouyei people. We observed that the contribution from Li-related ancestral sources ranged from 0.8860 to 0.9690. There was a low proportion of northern ancestry sources, which supported the idea that the Bouyei people originated from southern Chinese indigenous people and were affected by gene flow events from northern populations (Fig. 5F). Three-way qpAdm models also fit the admixture history of Bouyei (Fig. S12). Quantitative *f*-statistics demonstrated that Bouyei had a closer genetic relationship with the historical populations of Guangxi (Fig. 5C ∼ D). Therefore, we used the ancient northern YRB and southern rice farmers as surrogates and confirmed our hypothesis that the Bouyei populations could be modeled as north‒south ancestral admixtures (Fig. 5G). To further pinpoint the date of admixture events between northern and southern populations over a wide range of time, we conducted ALDER (Admixture-induced Linkage Disequilibrium for Evolutionary Relationship) analysis and discovered complex genetic admixtures in the Bouyei populations (Table S10). We found that admixture occurred in the BBN at approximately 55.86 ± 26.51 generations (1536 ± 742 CE) in the Han-Yao model and at 94.37 ± 35.94 generations (2614 ± 1006 CE) in the Han-Dongjia model.

### 2.4 Natural selection signatures and biological adaptation

**Fig. 6:**
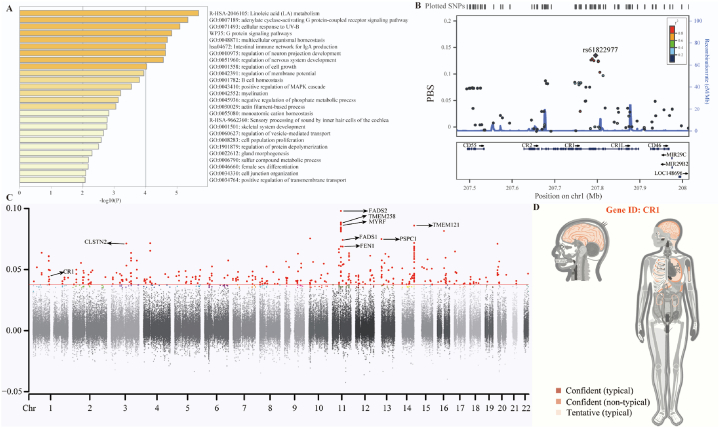
Positive natural selection signals and other relevant variations in the Bouyei populations. (**A**) Functional enrichment of the candidate genes for which the PBS values were in the top 100 according to Metascape online software (*Meta*-Bouyei-Shaanxi_Han-European). The enrichment p values showed genes associated with the pathway. (**B**) The corresponding adaptive variant (rs61229077), which was located in the *CR1* gene on chromosome 1, is marked, and other SNPs are colored based on pairwise linkage disequilibrium with the target variant. (**C**) Manhattan plot showing the PBS values from genome-wide scanning for the Bouyei population in Guizhou using the Miao_GZ and Shaanxi_Han populations as reference populations. The 99.9th percentile of the PBS distribution are shown as red lines. PBS values over the 99.9th percentile are marked in red, and PBS values under the 99.9th percentile are colored as dark dots. Otherwise, some of the genes were labeled with their names. (**D**) According to the Gene organizer, the CR1 gene affected relevant organs, systems, regions, and germ layers. (For interpretation of the references to color in this figure legend, the reader is referred to the Web version of this article.)

Our identified complex genetic admixture processes and environmental selection forces may contribute to the unique landscape of biological adaptative variants or genes in TK populations. We used multidimensional techniques based on the allele frequency spectrum and haplotype homozygosity to characterize the biological adaptative features of our first comprehensively reported TK lineage in Guizhou. First, we applied population branch statistics (PBS) with Northern Han Chinese individuals as the ingroup reference population and merged European individuals from HGDP genomic resources as the outgroup to identify the putative adaptative signatures that occurred in the Bouyei people after separation from Han Chinese individuals. Because of the substantial homogeneity of the Bouyei people, we combined four Bouyei groups into the merged *meta*-Bouyei population as the target population. Loci with PBS values greater than the 99.99th percentile were regarded as candidate selection variants. We identified 141 PBS-identified adaptative genes on chromosomes 1, 2, 3, 6, and 11 (Fig. S13, Table S11). We performed functional enrichment analysis based on the PBS-based signatures (Fig. 6A). The enrichment results revealed that the selection-related genes were associated with lipid metabolism [regulation of Linoleic acid (LA) metabolism (R-HSA-2046105)], immunity [Intestinal immune network for IgA production (hsa04672)], nervous development [regulation of nervous system development (GO:0051960)] and regulation of neuron projection development (GO:0010975)] and other development and proliferation pathways [multicellular organismal homeostasis (GO:0048871), G protein signaling pathways (WP35), cellular response to UV-B (GO:0071493), and adenylate cyclase-activating G protein-coupled receptor signaling pathway (GO:0007189)].

The highly noteworthy natural selection signals for Bouyei were identified in genes related to lipid metabolism and physical features, such as *FADS1* (fatty acid desaturase 1), *FADS2* (fatty acid desaturase 2), and *EDAR* (ectodysplasin A receptor). The strongest PBS selection signal was identified in *EDAR* (PBS = 0.3180), located on chromosome 2 (rs922452), which is a member of the tumor necrosis factor receptor family and plays a predominant role in hair straightness, skin tone, facial feature flatness, and sweat gland density in East Asians [31]. *FADS* is associated with lipid metabolism, as determined by long-chain polyunsaturated fatty acid (PUFA) levels, and mainly includes the *FADS1* and *FADS2* gene*s*. *FADS* encodes the fatty acid desaturase enzymes, which regulate the unsaturation of fatty acids by translating short-chain to long-chain PUFAs [30]. *FADS*-rs174570 had the greatest difference (PBS = 0.2533). Previous genetic studies have reported that *FADS* levels are associated with lipid metabolism disorder and hypobetalipoproteinaemia [[32], [33], [34]]. Ancient Southern Chinese people experienced a long history of infectious diseases. Recent modern and ancient European genomes have demonstrated that infectious disease risk increased since the early Neolithic period, and autoimmune-related disease risk and adaptative evolution occurred in the post-Bronze Age [35,36]. We also identified putative immune-related adaptative variants, and multiple variants were observed in *HLA-DPA1* (Major Histocompatibility Complex, Class II, DP Alpha 1) and *CR1* (Complement C3b/C4b Receptor 1, Knops Blood Group). Among the polypeptides produced by extracellular proteins, *HLA-DPA1,* a protein-coding gene, mainly plays a crucial role in the immune system. Key mutations in *HLA-DPA1* are associated with genetic susceptibility to rare autoimmune diseases, such as granulomatosis with polyangiitis [32,33]. Epidemiological data have revealed a high incidence of Malaria in South China, and southern Chinese people exhibit adaptive features during persistent pathogen exposure. *CR1* is vital for mutual effects within P. falciparum and interrelated hosts at different levels. Genetic variations in *CR1* lead to *CR1* deficiency, which occurs in regions with high incidences of Malaria, yet this mechanism can prevent severe Malaria. In detail, parasitized red blood cells (RBCs) invaded by severe Malaria typically adhere to complement receptors located on other uninfected RBCs and play corresponding physiological roles in forming clumps of cells that are also referred to as rosettes [[31], [32], [33],[37], [38], [39], [40], [41]]. In addition, we observed that rs61822977, which has the highest PBS value on chromosome 11, exhibited robust biological adaptation and associations with other identified *CR1*-related SNPs. In addition, we generated a regional plot for the top-ranked SNP rs61822977 and other nearby variants in 200 kb based on LD. Other immune-related adaptative variants (*CD55*, *CR2*, *CR1L* and *CD46*) were also marked (Fig. 6B). However, because of the complexity of host-parasite interactions, more research is needed to elucidate the vital molecular markers and corresponding biomedical mechanisms involved in the pathogenesis of Malaria.

**Fig. 7:**
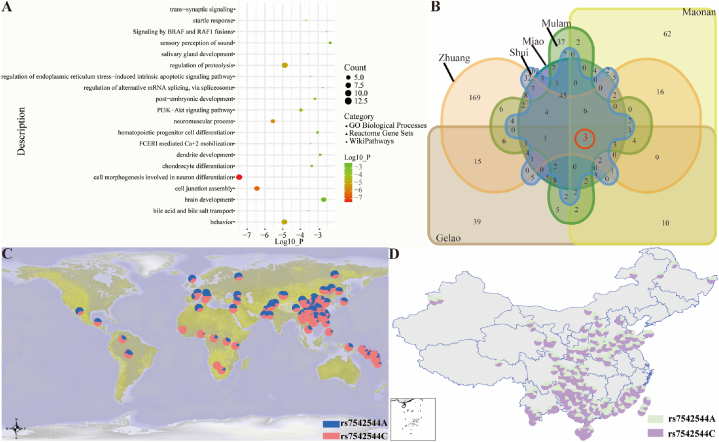
Regional-specific natural selection signals. (**A**) Functional enrichment of the candidate genes whose PBS values were greater than the 99.9th percentile according to the Metascape online software (*Meta*-Bouyei-Miao_GZ-Shaanxi_Han). Additionally, these candidates formed the top 20 clusters based on three categories: "Count" was the number of genes in these candidates for each cluster, "Log10" was the p-value in log base 10, and a − log10_(P value)_ > 2 indicated that the functional category was considered significant. (**B**) The Venn diagram represents the overlaps of positive natural candidates in six PBS groups (*Meta*-Bouyei-Miao/Gelao/Zhuang/Shui/Mulam/Maonan-Shaanxi_Han). (**C**) The frequency distribution of *CR1* (rs7542544 A/C) based on the 10K_CPGDP database. (**D**) The frequency distribution of *CR1* (rs7542544 A/C) based on East Asian populations from the 10K_CPGDP database. 10K_CPGDP: 10K Chinese People Genomic Diversity Project.

To determine the differences in regional-specific natural adaptation signals of the Bouyei people, we also calculated the PBS using neighboring Miao and Northern Han people as the ingroup and outgroup, respectively. We identified 132 adaptative genes within the candidate selection variants above the 99.99th percentile according to the PBS values (Fig. 6C–Table S12). In addition to genes such as *FADS1*, *FADS2,* and *CR1*, we also found strong candidate genes related to familial diseases. For example, *CLSTN2* (calsyntenin 2) lies on chromosome 3 and is relevant to astigmatism; *TMEM258* (transmembrane protein 258) is related to spinocerebellar ataxia; another gene, *TMEM121* (transmembrane protein 121), plays an essential role in the membrane; and *FEN1* (flap structure-specific endonuclease 1) is linked to Xeroderma pigmentosum through base excision repair [32,42]. The Myelin Regulatory Factor (*MYRF*) is located on chromosome 11 and encodes a transcription factor essential for the development of the myelin central nervous system and myelination [43]. It can be regarded as increasing gene expression and further influencing myelin production, while others directly facilitate myelin gene expression. In addition, for the *CR1* gene, which was also identified in regional-specific analyses, we used the Gene ORGANizer (http://geneorganizer.huji.ac.il/) to explore the association between the *CR1* gene and organ systems. We observed that our identified candidate adaptative genes were potentially linked and further influenced by our brain and lungs, corresponding to the physiological mechanism related to the Malaria pathogens that invade and damage the human body (Fig. 6D). Using the enrichment database in Metascape, we found that these candidates were related to essential physiological functions of the human body, such as cell‒cell adhesion, neuronal system, development process, and immune system process. In addition, we performed pathway and process functional enrichment with three ontology sources, GO Biological Processes, Reactome Gene Sets, and WikiPathway, to investigate the detailed functional connections. Among the 132 candidates under natural selection, 20 functional groups were identified (Fig. 7A, Table S13). The functional category was considered significant when the log10_(P- value)_ was greater than 2. Cell morphogenesis involved in neuron differentiation (GO:0048667) and regulation of proteolysis (GO:0030162) accounted for the greatest proportion (approximately 10.08 %). We also calculated the five PBS values using different ethnic minorities in Guizhou, including Zhuang/Shui/Mulam/Maonan and Gelao as the ingroup and Northern Han as the outgroup (Fig. 7B), to detect the specific signals of adaptive evolution among the Bouyei population. Except for two genes (*CLSTN2* and *CR1*) mentioned above, the *PTPRD* (protein tyrosine phosphatase receptor type D) gene, which is a member of the protein tyrosine phosphatase (PTP) family and is relevant to Restless Legs syndrome and chromosome 9P deletion syndrome, was identified in all six analyses. In six analyses of different ethnic groups in Guizhou, the *CR1* gene was screened simultaneously, verifying that *CR1* plays a vital role in resistance to Malaria in Guizhou. Therefore, based on the 10K Chinese People Genomic Diversity Project (10K_CPGDP) database, we explored the distribution of allele frequencies of *CR1-*related mutations (rs7542544) on diverse scales (Fig. 7C) and focused on the frequency distribution in China, especially in southern China (Fig. 7D). We observed that this mutation (rs7542544) presented an intense natural selection signal, and its derived haplotype was highly expressed in southern China, which also conformed to the frequency distribution in Sardinia, Papua New Guinea, Bantu_Kenya and other Malaria-endemic regions [38]. Notably, this phenomenon of high frequencies of the derived allele also occurred in TK-related populations with different geographical environments, which further indicated that their common ancestors were affected by strong natural selection, followed by population admixture, migration, genetic drift and bottleneck events. A typical example was the frequency distribution pattern in Hainan Li, which can be representative of the coastal TK-speaking population [18]. Furthermore, eight other loci also confirmed the prevalence of Malaria among TK-speaking populations: seven of nine variants (rs7542544, rs11576522, rs12036785, rs61822977, rs11803956, rs12041437, and rs12034383) were considered as candidate selection variants, and the other two variants (rs11803366 and rs10779339) were CR1-related mutations (Table S14). Consequently, RBC *CR1* deficiency caused by high expression of *CR1*-derived alleles is profoundly common in southern China malaria-endemic regions such as Guizhou and other SEAs. Polymorphisms associated with *CR1* deficiency confer protection against severe Malaria [38].

## 3 Discussion

### 3.1 Demographic history and population structure within TK-speaking populations

Rice farming originated in the Yangtze River and led to the development of southern Chinese TK/HM/AA/AN-related populations, which play an essential role in population migration southward to SEA [8,32,44,45]. The ancient "Baiyue" living in Southeast China were considered to be the ancestors of the present-day TK-speaking populations. The TK-speaking populations were the indigenous people of southern China. Continuous population admixture and isolation events contributed to the specific genetic structure and evolutionary history of these populations. Previously, few studies have highlighted inland and integrated groups, and more research has focused on the genetic structure of single and coastal groups. One of the typical representatives was Hainan Li, which has a relatively isolated genetic background and complex evolutionary history [15]. Therefore, the genetic origin and phylogenetic relationships within TK-speaking populations remain to be further characterized [34,[46], [47], [48]]. We collected and genotyped 24 BBN individuals and merged newly generated data with previously published data to produce three datasets—a low-density merged HO dataset [28,29], a middle-density merged 1240K dataset, and a high-density merged WGS dataset [[49], [50], [51], [52], [53], [54], [55]] to provide new insights into the demographic history, population structure, and human genetic diversity of TK-speaking individuals. The ADMIXTURE result based on a high-density dataset identified a unique genetic component that was greatest in TK-speaking Bouyei, indicating that the Bouyei populations possessed a unique genetic structure (Fig. 1A). As the high-density dataset lacked SNP data on other TK-speaking populations from MSEA, we further included populations genotyped via the Affymetrix Human Origin array and explored the general patterns of genetic structure among 39 TK-speaking populations and their relationships with other ancient/modern humans (Fig. 1B∼2). Our results showed that TK-speaking populations from MSEA and China had similar genetic structures and close genetic affinities (Fig. 2B ∼ C and 3). We identified the population's genetic substructure related to their geographic distribution patterns. The 39 TK-speaking populations could be roughly divided into two clusters: southern TK-speaking populations from MSEA and northern TK-speaking populations from China, which showed apparent genetic differentiation. The Bouyei group and the surrounding TK-speaking population (Maonan population) clustered together and exhibited increased allele sharing. The TK-speaking populations from MSEA showed similar genetic structures and relationships.

Guizhou Province is located in the Yungui Plateau and has mountainous topographical features, resulting in an isolated geographical environment and a unique genetic landscape [25]. By concentrating on 14 populations in areas with high ethnic and linguistic diversity based on a high-density dataset, we identified their high genetic affinity and similar genetic origins (Fig. 4). We used admixture *f*_3_ and GLOBETROTTER to detect the admixture signatures of the BBN population (Fig. 5A). Both genetic alleles and haplotype blocks confirmed that Bouyei has a unique evolutionary history. Moreover, a high degree of genetic homogeneity within four Bouyei groups and widespread heterozygosity within TK-speaking populations were demonstrated through the observed results of *f*_4_(Studied1, Studied2; Reference, Mbuti) and qpWave (Figures S10 and 11). In addition, a previous study illustrated that 1500-year-old BBQC people in Guangxi were the direct ancestral source of the modern TK people. We observed that the estimates of *f*_4_(BBQC, Bouyei; Reference populations, Mbuti) were consistent with those of previous studies. The Bouyei populations shared more alleles with BBQC and were influenced by gene flow events from neighboring minorities (Fig. 5C ∼ D). The results for *f*_4_(Reference population1, Reference population2; Bouyei, Mbuti) also revealed widespread gene flow events from surrounding ethnic groups (Fig. 5B). Finally, the qpAdm-based two/three-way admixture models and ALDER further confirmed their potential admixture proportions and times (Fig. 5F ∼ G). The Bouyei populations originated from southern indigenous people and were affected by gene flow events from northern populations.

### 3.2 Local adaptation of guizhou populations

To understand the genetic basis of local adaptation signatures in the Bouyei population, we performed PBS analyses with Northern Han Chinese individuals as the ingroup and European_HGDP individuals as the outgroup and identified 141 adaptative genes with values above the 99.99th percentile (Table S8). According to the functional enrichment analysis, we observed different signaling pathways, including immune, lipid metabolism, and physical traits (Fig. 6A). *HLA-DPA1* mainly plays a central role in the immune system by presenting peptides derived from extracellular proteins [32,33]. *FADS1* and *FADS2* mainly encode fatty acid desaturase enzymes and control the unsaturation of fatty acids [30]. The *EDAR* gene is typically associated with East Asian features, including the shovel shape of upper incisors, hair straightness, and facial characteristics [[56], [57], [58], [59]]. The physiological functions associated with *CR1* mainly include opsonization*,* control of complement activation, and removal of immune complexes (ICs). *CR1* deficiency was also related to Malaria resistance, which can greatly reduce the resetting of the rosette. Specifically, RBCs invaded by severe Malaria typically adhere to complement receptors located on other uninfected RBCs and play corresponding physiological roles in rosette formation, causing severe obstruction of the cerebral microvasculature and thus resulting in pathological changes in cerebral malaria [38]. Furthermore, we constructed a regional plot for the top-ranked SNP rs61822977 and found nearby immune-related putative adaptive variants (*CD55*, *CR2*, *CR1L,* and *CD46*) in 200 kb based on LD (Fig. 6B). We also calculated the PBS using the neighboring Miao and northern Han people as the ingroup and outgroup, respectively, to explore the differences in region-specific natural adaptation signals within the Bouyei people. We identified 132 adaptative genes within the candidate selection variants over the 99.99th percentile (Fig. 6C–Table S9), which were related to essential physiological functions of the human body through the enrichment database in Metascape (Fig. 7A–Table S10). To further detect the specific signals of natural selection within the Bouyei populations, we used the Zhuang/Shui/Mulam/Maonan and Gelao populations in Guizhou as the ingroup and the Northern Han population as the outgroup to calculate the other five PBS values (Fig. 7B). Interestingly, the *CR1* gene was screened simultaneously, suggesting that *CR1* plays a vital role in resistance to Malaria in Guizhou. In addition, we identified other strong candidate genes, such as *CLSTN2, TMEM258, TMEM121, FEN1,* and *MYRF*, which play different roles in relevant organs and systems, in addition to genes such as *FADS1*, *FADS2,* and *CR1.* For example, *MYRF* is a well-known gene located on chromosome 1 that encodes a transcription factor required for central nervous system myelination [43]. Combined with the frequency distribution of nine related loci based on the 10K_CPGDP database, we found that the derived haplotype of *CR1* was strongly selected and highly expressed in southern China, such as in Guizhou and MSEA, resulting in Malaria resistance in endemic areas (Fig. 7C ∼ D) [38].

Guizhou has been described as a "miasmatic region" with endemic diseases such as Malaria epidemics that seriously affect the health of the local population and further influence social and economic development [60]. Before liberation, the annual number of Malaria cases in Guizhou Province was between 2 million and 3 million, representing 25–30 % of the total population. For decades, the government has vigorously pursued a comprehensive prevention and control strategy focusing on controlling the source of infection. However, with the increasing frequency of global economic, mass travel, and business affairs abroad from Malaria-endemic countries and regions, the threat of imported Malaria will continue to exist in Guizhou Province. The formation of ICs, which can generate proinflammatory cytokines and thus stimulate macrophages and monocytes, is a prominent feature of Malaria infection, and *CR1* also plays a crucial role in IC clearance due to its high affinity for C3b and C4. Erythrocyte *CR1* (*E*-*CR1*) binds ICs in the peripheral blood through "immune adherence", which can transport them to phagocytes in the liver or spleen to further remove them from the circulation. Other research also showed that erythrocytes with high levels of *CR1* carry a greater quantity of ICs, which stimulates the production of proinflammatory cytokines, thus increasing the incidence of cerebral malaria. Therefore, the *CR1* levels on erythrocytes and relevant polymorphisms have been associated with the response to P. falciparum Malaria in Malaria-endemic regions. Moreover, the severity of P. falciparum malaria has been linked to several human genetic factors, such as sickle cell disease, thalassemia, and G6PD deficiency. These diseases caused by erythrocytic defects are considered the selective pressure behind them.

## 4 Conclusion

We newly collected 24 BBN samples from Guizhou and merged them with publicly available genome-wide SNP data from 38 TK-speaking populations to form one aggregated dataset. We explored the population structure, evolutionary history, and biological adaptation of 39 geographically different TK-speaking populations. We found that the Bouyei groups were genetically similar to neighboring TK-speaking populations. Furthermore, due to its unique genetic structure, Bouyei-related ancestry can be used as an optimal representative of inland TK-related populations. The shared haplotypes and alleles showed genetic heterozygosity among 39 TK people from diverse geographical environments. In addition, we found that 39 TK-speaking populations exhibited prominent geography-related population substructures, and the clustering patterns were associated with their extensive differences in genetic diversity. We found that four Bouyei populations had substantial genetic homogeneity, simulated admixture time, and constructed admixture models within the populations, further confirming their distinct genetic origins. We also identified north-to-south admixture events consistent with genetic affinity and historical population movements. It was confirmed that Bouyei could be modeled as an admixture of minor northern Chinese populations and major southern Chinese populations, and ancient southern Chinese people constituted the majority of the Bouyei ancestry. We identified several candidates through population and regional-specific analyses, including the *CR1* gene, which is associated with immunity and Malaria resistance, and other genes involved in metabolic evolution.

## 5 Materials and methods

### 5.1 Sample collection and DNA preparation

We collected saliva samples from 24 unrelated Bouyei individuals from Bannong County in Guizhou Province (Fig. S14). The participants' parents and grandparents were indigenous people residing in the sampling palaces for at least three generations. All participants had nonconsanguineous marriages in the same ethnic group. The Medical Ethics Committees of West China Hospital of Sichuan University (2023-306) approved this study. In addition, the procedure followed the recommendations of the Helsinki Declaration as revised in 2000. All the participants signed written informed consent before participating in the study. We extracted human genomic DNA with a PureLink Genomic DNA Kit (Thermo Fisher Scientific) and measured the concentration with a Nanodrop 2000. In addition, we genotyped the above samples using Illumina arrays of approximately 700K SNPs.

### 5.2 Relatedness analysis

We used KING2 to test individual relationships by calculating kinship coefficients before merging the data. All unrelated samples were kept for the following analyses.

### 5.3 Data merging

We used PLINK v.1.9 to obtain the quality-controlled raw data from the generated data and then combined it with publicly available data. We identified and filtered the missing SNPs and individuals with the parameters of --geno 0.05 and --mind 0.05. We merged our genotyped Bouyei data with 38 publicly available and previously published TK-speaking populations from the Southern China and SEA datasets, forming the primary TK-related dataset to explore the population structure and genetic relationships of whole TK-speaking populations [3,34,45,53,61]. The TK dataset included 796 publicly available individuals from 38 populations, of which 522 were from southern China and 274 were from SEA. In addition, to explore the genetic relationship between TK people and other modern and ancient eastern Eurasians, we merged our genome-wide data with previously published modern and ancient people from the Allen Ancient DNA Resource (AADR), which formed a low-density merged HO dataset [28,29], including 56,814 SNPs, and a middle-density merged 1240K dataset, including 146,802 SNPs. To explore the fine-scale genetic structure and obtain phased genomes to illuminate the biological adaptation mechanisms, we combined our data with previously published Illumina data [[49], [50], [51], [52], [53], [54], [55],^62^], 54 worldwide populations included in the HGDP [28], and 20 populations from Taiwan Island, SEA Island, and Oceania included in the Oceanian genomic resources [29], which formed the high-density merged WGS dataset and included 460,678 SNPs.

### 5.4 Principal component analysis

Using the merged HO dataset, we carried out PCA via the smartPCA program of the EIGENSOFT v.6.1.4 package [61]. All default parameters were used with the additional parameters of the LSQ project: YES. In addition, we used PLINK to prune the data with the parameters "-indep-pairwise 200 25 0.4" [55]. We performed three levels of PCA, focusing on the relationships between different regional East Asian populations and TK-speaking populations. PCA was first performed using 207 modern and ancient East Asian populations to explore their genetic similarities and differentiations [63]. Second, modern East Asian populations were extracted for further intraregional PCA. The ancient samples from the YRB, AR, West Liao River, Guangxi, Fujian, Taiwan Island, and other neighboring regions were projected on the PCA of contemporary East Asians [64]. We explored the genetic affinity of the studied and other ethnic groups among 117 southern reference populations. Finally, we performed a fine-scale analysis of 39 TK-speaking populations.

### 5.5 Model-based ADMIXTURE analysis

The model-based maximum likelihood clustering algorithm implemented in the ancestry estimation method ADMIXTURE [65] was applied to explore the genetic composition of TK-related populations using the merged Illumina, HGDP, and Oceanian datasets of 3514 individuals from 207 modern worldwide populations and the low-density merged HO dataset including 117 southern Chinese populations. We used ADMIXTURE mainly to identify Bouyei's genetic structure and estimate individuals' ancestry in the context of worldwide and regional people. We used PLINK v.1.0711 [55] to prune the original dataset with dense SNPs before analyzing the ADMIXTURE. We ran ADMIXTURE 100 times with default parameters and the number of ancestral populations from K = 2 to K = 20 in bootstrap sequences with different random seeds [65]. We selected an optimal K value (K = 11) based on the lowest cross-validation error and the highest log-likelihood using 10-fold cross-validation with different random seeds [66]. We selected an optimal K value (K = 3) at the regional scale to explore the ancestry composition within the Bouyei groups and other TK-related populations in Guizhou based on the high-density merged WGS dataset.

Moreover, we performed an ADMIXTURE analysis of 117 modern and ancient samples based on a low-density dataset to explore the genetic structure in an ancient context; when K = 6, the cross-value was the lowest. We selected an optimal K value (K = 4) at the regional scale to explore the ancestry composition within the Bouyei groups and other TK-related populations from different geographical locations based on the low-density merged HO dataset. In addition, we combined the population phylogenetic topology within 117 southern Chinese populations based on the values of 1-outgroup-*f*_3_ and model-based ADMIXTURE results (K = 6). We further explored the genetic differentiation and substructure within 39 TK populations at the individual level using haplotype-based fineSTRUCTURE and admixture model reconstructions based on the allele frequency spectrum when we selected K = 4 as the optimal value. Similarly, we also used the same analysis for the 14 groups in Guizhou based on the high-density merged WGS dataset when we selected K = 3 as the optimal value.

### 5.6 Pairwise F_ST_ genetic distances

The pairwise fixation index (F_ST)_ was estimated to explore the genetic affinity among geographically different populations based on the high-density dataset using PLINK v1.90 [5]. We also calculated the pairwise F_ST_ genetic distance to measure the genetic relationship between Guizhou and SEA TK-speaking populations based on the low-density dataset using PLINK v1.90.

### 5.7 TreeMix analyses

We constructed a TreeMix-based phylogenetic tree of 14 populations to infer the genetic relationships and evaluate the gene flow events between BBN and other TK-speaking populations [67]. Using the Illumina dataset array, a phylogenetic tree with migration events varying from 0 to 2 was reconstructed to study the genetic patterns of population split and admixture between our target and multiple ancestral populations. We also constructed a TreeMix-based phylogenetic tree within 39 geographically diverse groups to explore the genetic affinity when two gene flow events occurred.

### 5.8 Runs of homozygosity (ROH)

We estimated the indicator of genomic homozygosity within 39 TK-speaking populations based on the low-density dataset using PLINK v1.90 [55]. We used ROHs containing at least 50 SNPs and consecutive SNPs more than 100 kb apart, which were regarded as independent ROHs. Finally, we statistically visualized the ROH distribution of each TK-speaking population via box plots using R version 3.5.2. Furthermore, we explored the length distribution patterns of ROHs within TK people in Guizhou based on a high-density dataset.

### 5.9 IBD estimation and effective population size inference

The IBD blocks were divided into three categories: <1, 1–5, and >5 cM, which correspond very roughly to time intervals of early events (approximately 1500–2500 years ago), interim events (approximately 500-1500 years ago), and recent events (approximately 0–500 years ago), respectively. Considering the impact of the noise signal, we eliminated the smallest IBD segments, which reflected ancient events. Hence, we generated only two catalysts, 1–5 and >5 cM, using Refined-IBD software (16May19. ad5. jar) with a length parameter of 0 [68]. Ne was used to estimate the effective population size among geographically diverse Bouyei people.

### 5.10 Admixture-*f*_*3*_-statistics and outgroup-*f*_3_-statistics

We used ADMIXTOOLS software [69] to compute *f*-statistic values and estimate standard errors by a block jackknife and default parameters. First, we explored the potentially existing admixture signals within Bouyei and other modern/ancient populations in East Asia and performed admixture-*f*_3_-statistics in the form of *f*_3_(Source 1, Source 2; Targeted population) through qp3pop to confirm whether the studied population was admixed. A target population with a negative *f*_3_ value and a value of approximately |Z score|>3 was regarded as a potential admixture signal. Since the *f*_*3*_-based estimates produce statistically significant values, we lowered the threshold, set the |Z score|>2, and visualized the top 20 values used in R packages. Second, we selected modern East Asian and ancient populations as reference populations and performed the outgroup-*f*_3_ statistics in *f*_3_(Reference, Studied populations; Mbuti) via the qp3Pop program of EIGENSOFT to explore the genetic affinity and drift between the studied populations and other reference populations. Here, we conducted four group analyses, including analyses of 39 TK-speaking populations, 37 TK-speaking populations (not containing CentralThai and SouthernThai_TK), 42 ancient populations, and 38 Guizhou populations, and constructed a heatmap using R packages. A higher value and darker color indicate a closer relationship.

### 5.11 *F*_4_-statistics

We conducted four population tests for targeted people based on the individual sample and merged populations [69]. We calculated the *f*_4_-statistics to explore the signals and directions of admixture and the primary source of gene flow to the Bouyei populations and other modern and ancient reference populations. Modern reference populations from the low-density dataset were used to test for genetic differences among geographically different Bouyei people; ancient reference populations from the middle-density dataset were used to further verify the accuracy of the *f*_*4*_-statistics mentioned above. Therefore, we used qpDstat in ADMIXTOOLS to conduct the *f*_4_(Studied1, Studied2; Reference populations, Mbuti) to explore the genetic heterozygosity and homogeneity among the studied groups and then visualized the top 62 values using R packages. Then, we performed *f*_4_-statistics in the form of *f*_*4*_(Reference population1, Reference population2; BBN, Mbuti) to test the patterns of shared alleles between the BBN and reference populations. Reference population 1 included eight ancient YRB farmers from northern China, namely, China_NEastAsia_Coastal_EN, Upper_YR_LN, Shimao_LN, YR_MN, YR_LN, and YR_LBIA, and Reference population2 included ancient people from Guangxi, Fujian, and SEA. When focusing on modern people, the reference populations included AA/AN/TK/HM-related southern populations and other northern populations. Additionally, we merged four geographically different Bouyei people as one integrative Bouyei population (*Meta*-Bouyei) to search for optimum ancestry sources at the population level in the form *f*_4_(Reference populations, *Meta*-Bouyei; BBQC, Mbuti) and in the form *f*_4_(BBQC, *Meta*-Bouyei; Reference populations, Mbuti). The reference populations included ancient people from different historical periods.

### 5.12 Pairwise qpWave and qpAdm estimation

We used qpAdm [70] implemented in the ADMIXTOOLS package to estimate the corresponding admixture proportions quantitatively with default parameters. We used ancient northern East Asians as the northern surrogate and southern East Asians as the southern surrogate to model the formation of modern Bouyei people via qpAdm. We simulated the modern admixture model using Northern Han, Tu, Hezhen, Xibo, Mongolian, Daur, and Oroqen as the northern sources and Li as the southern source. We used Yakut, Mbuti, Iran_GanjDareh_N, Villabruna, Ami, Mixe, Onge, Papuan and Ust_Ishim as basic outgroups. Moreover, we applied the ancient admixture model using Miaozigou_MN, NEastAsia_Coastal_EN, NEastAsia_Inland_EN, Shimao_LN, Upper_YR_LN, WLR_LN, YR_LN, YR_MN, and WLR_MN as the northern sources and China_SEastAsia_Island_LN, Taiwan_Hanben_IA, Yiyang, and Layi as the southern sources, respectively.

We also conducted pairwise qpWave analysis among modern TK/HM/AA/AN/TB, Sinitic, and Tungusic people based on the merged HO dataset to explore their genetic homogeneity within different pairwise populations and geographic scales. "+" indicates p_rank0 > 0.01 and was considered to indicate statistical significance, and "++" indicates p_rank0 > 0.05. We also performed pairwise qpWave analysis to explore the genetic homogeneity and heterogeneity within the Bouyei population.

### 5.13 Admixture time estimation with ALDER analysis

Admixture and migration can result in the exponential decay of LDs. Hence, we selected multiple northern and southern modern populations in the context of East Asia as potential ancestral sources and used MALDER to examine the admixture LD decays, estimate the generations, and explore the significant admixture signatures of the Bouyei people in Guizhou. We also tested all possible source combinations with the default parameters of mindis: 0.005 in Morgan and leave-one-chromosome-out (jackknife: YES) [71].

### 5.14 CHROMOPAINTER and fastGLOBETROTTER

We used ChromoPainterv2 to determine the ancestral haplotype compositions of surrogates and four Bouyei populations. In this case, we ran fastGLOBETROTTER based on the default parameters to identify, describe, and date the admixture events [72].

### 5.15 Painting chromosomes and fineSTRUCTURE analysis

We used SHAPEIT v2 (Segmented Haplotype Estimation & Imputation Tool) software with the default parameters (--burn 10 --prune 10 --main 30) to phase the genome-wide data of four Bouyei populations in Guizhou and ten other populations in geographically neighboring regions based on the high-density merged WGS dataset and 35 other TK-speaking populations based on the low-density merged HO dataset [73]. Then, to dissect the fine-scale population stratifications, we used ChromoPainter to compute the shared haplotypes and obtain the coancestry matrix [74]. Moreover, we used R packages implemented in fineSTRUCTURE and performed admixture analysis to explore the phylogenetic relationships and fine-scale structure of the Bouyei populations [72].

### 5.16 Signatures of natural selection

We applied PBS to detect population- and region-specific natural selection signals in the Bouyei populations [75]. First, we used Northern Han (Shaaxi_Han) individuals as an ingroup and European individuals as an outgroup to explore ancient natural selection signals. Second, we explored regional natural selection signals using Bouyei_Guizhou as the target population and Zhuang/Shui/Mulam/Maonan/Gelao in Guizhou and Shaaxi_Han as the ingroup and outgroup, respectively. The top 0.1 % of the PBS calculations were considered as candidates, and the PBS calculation formula was PBS_A_ = (T_AB_ + T_AC_−T_BC_)/2, T = −log(1−F_ST_), where A was the target population and B and C were the ingroup and outgroup populations, respectively. In addition, we calculated the allele frequency of selected alleles and mapped the frequency distribution globally based on our 10K_CPGDP.

### 5.17 Functional annotation of natural selection signatures

In two population- and region-specific analyses, we identified 141 and 132 candidate variants, respectively, with PBS values in the top 0.1 %. To search for candidates associated with different pathways in humans, we selected these candidates as the input gene set to perform functional enrichment by Metascape (https://Metascape.org), which incorporates numerous functional categories and is beneficial for further analysis [76]. This analysis used the following ontology sources to perform functional enrichment: GO Biological Processes, Reactome Gene Sets, and WikiPathway. The top 20 functional categories with a −log10_(P-value)_ ≥2 were considered enriched pathways.

## Data availability

The allele frequency data derived from human samples have been deposited in the National Omics Data Encyclopedia (NODE, http://www.biosino.org/node). The access and use of the data complied with the regulations of the People's Republic of China on the administration of human genetic resources. The results of the analyses in this study have been submitted to the supplementary materials and deposited into the OMIX database (https://ngdc.cncb.ac.cn/omix/) through accession number OMIX005449. Requests for access to data can be directed to Guanglin He (Guanglinhescu@163.com).

## Ethics approval and consent to participate

The Medical Ethics Committees of West China Hospital of Sichuan University approved this study. The principles of the Helsinki Declaration were used to conduct this study.

## Consent for publication

Not applicable.

## CRediT authorship contribution statement

**Shuhan Duan:** Writing – review & editing, Writing – original draft, Visualization, Validation, Supervision, Software, Project administration, Methodology, Investigation, Formal analysis, Data curation, Conceptualization. **Mengge Wang:** Writing – review & editing, Writing – original draft, Funding acquisition, Data curation, Conceptualization. **Zhiyong Wang:** Writing – review & editing, Writing – original draft, Visualization. **Yan Liu:** Resources, Data curation. **Xiucheng Jiang:** Supervision, Software. **Haoran Su:** Methodology, Conceptualization. **Yan Cai:** Software. **Qiuxia Sun:** Software. **Yuntao Sun:** Software. **Xiangping Li:** Validation, Software. **Jing Chen:** Visualization. **Yijiu Zhang:** Software. **Jiangwei Yan:** Validation. **Shengjie Nie:** Visualization. **Liping Hu:** Software. **Renkuan Tang:** Validation. **Libing Yun:** Visualization. **Chuan-Chao Wang:** Visualization. **Chao Liu:** Visualization. **Junbao Yang:** Writing – review & editing, Writing – original draft. **Guanglin He:** Writing – review & editing, Writing – original draft, Visualization, Validation, Supervision, Software, Resources, Project administration, Methodology, Investigation, Formal analysis, Data curation, Conceptualization.

## Declaration of competing interest

The authors declare the following financial interests/personal relationships which may be considered as potential competing interests:Mengge Wang reports financial support was provided by 10.13039/501100013365West China Hospital of 10.13039/501100004912Sichuan University. If there are other authors, they declare that they have no known competing financial interests or personal relationships that could have appeared to influence the work reported in this paper.
