# Supplementary material for: Malaria resistance-related biological adaptation and complex evolutionary footprints inferred from one integrative Tai-Kadai-related genomic resource
Source: Heliyon. 2024 Apr 9;10(8):e29235. doi: 10.1016/j.heliyon.2024.e29235 (PMC11043949; doi:10.1016/j.heliyon.2024.e29235)
Supplement: Multimedia component 2 [file mmc2.docx]

**Supplementary Figures for:**

**Malaria resistance-related biological adaptation and complex evolutionary footprints inferred from one integrative Tai-Kadai-related genomic resource**

Shuhan Duan^1,2*^, Mengge Wang^8,9*,#^, Zhiyong Wang^2,6*^, Yan Liu^1,2*^, Xiucheng Jiang^1,2^, Haoran Su^1,2^, Yan Cai^1,3^, Qiuxia Sun^2,4^, Yuntao Sun^2,7^, Xiangping Li^2,6^, Jing Chen^2^^,5^, Yijiu Zhang^2,4^, Jiangwei Yan^5^, Shengjie Nie^6^, Liping Hu^6^, Renkuan Tang^4^, Libing Yun^7^, Chuan-Chao Wan^8^, Chao Liu^9#^, Junbao Yang^1,3*,#^, Guanglin He^1,2,3,10,#^

^1^Institute of Basic Medicine and Forensic Medicine, North Sichuan Medical College and Center for Genetics and Prenatal Diagnosis, Affiliated Hospital of North Sichuan Medical College, Nanchong, Sichuan, 637007, China ^2^Institute of Rare Diseases, West China Hospital of Sichuan University, Sichuan University, Chengdu, 610000, China

^3^Research Center for Genomic Medicine, North Sichuan Medical College, Nanchong, 637100, China

^4^Department of Forensic Medicine, College of Basic Medicine, Chongqing Medical University, Chongqing, 400331, China

^5^School of Forensic Medicine, Shanxi Medical University, Jinzhong, 030001, China

^6^School of Forensic Medicine, Kunming Medical University, Kunming, 650500, China

^7^West China School of Basic Science & Forensic Medicine, Sichuan University, Chengdu, 610041, China

^8^State Key Laboratory of Cellular Stress Biology, National Institute for Data Science in Health and Medicine, School of Life Sciences, Xiamen University, Xiamen 361005, Fujian, China

^9^Anti-Drug Technology Center of Guangdong Province, Guangzhou, 510230, China

^10^Center for Archaeological Science, Sichuan University, Chengdu, 610000, China

*Shuhan Duan, Zhiyong Wang, Junbao Yang, Yan Liu, and Mengge Wang contributed equally to this work

^#^Correspondence: Mengge Wang ([Menggewang2021@163.com](mailto:Menggewang2021@163.com)), Chao Liu ([liuchaogzf@163.com](mailto:liuchaogzf@163.com)), Junbao Yang (yjb3589@vip.163.com), Guanglin He ([guanglinhescu@163.com](mailto:guanglinhescu@163.com))

**
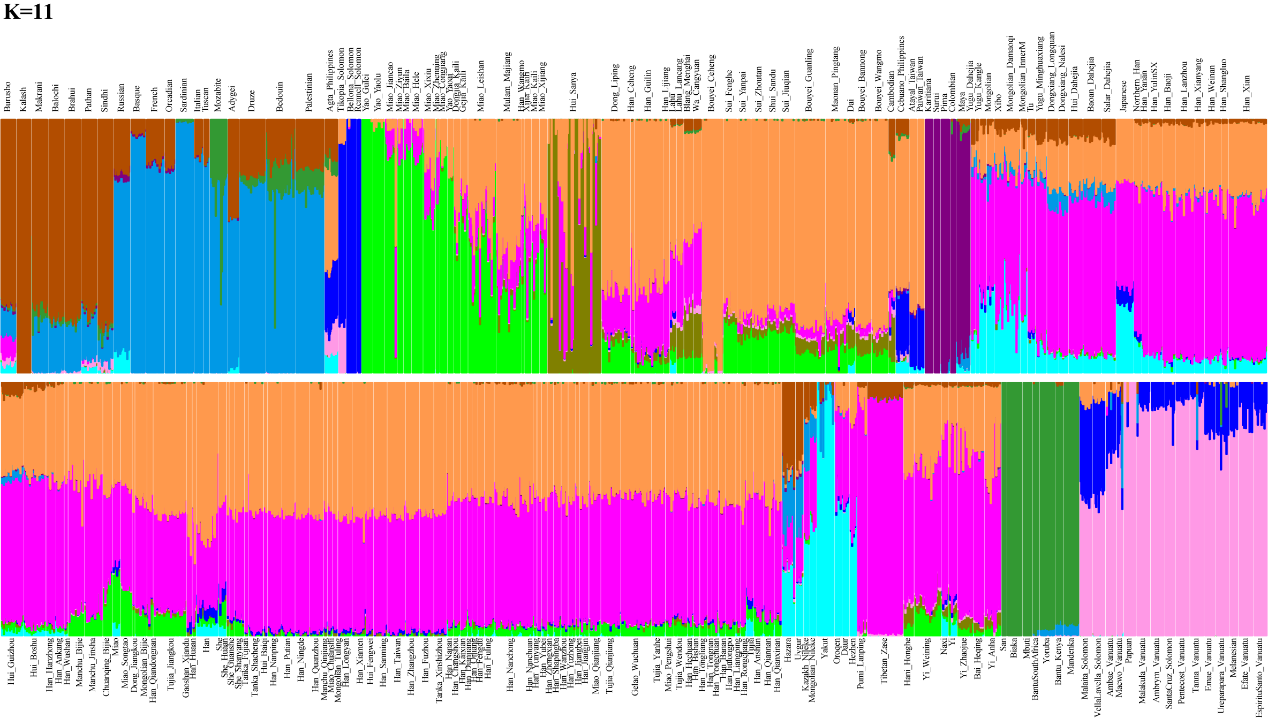
**

**Figure S1. General population structure among different modern and ancient populations.**

Results of model-based ADMIXTURE clustering analysis. Based on a high-density dataset, the clustering patterns were visualized with the 207 populations at K=11. Different colors presented different ancestral components. The width of the column is related to the sample size.


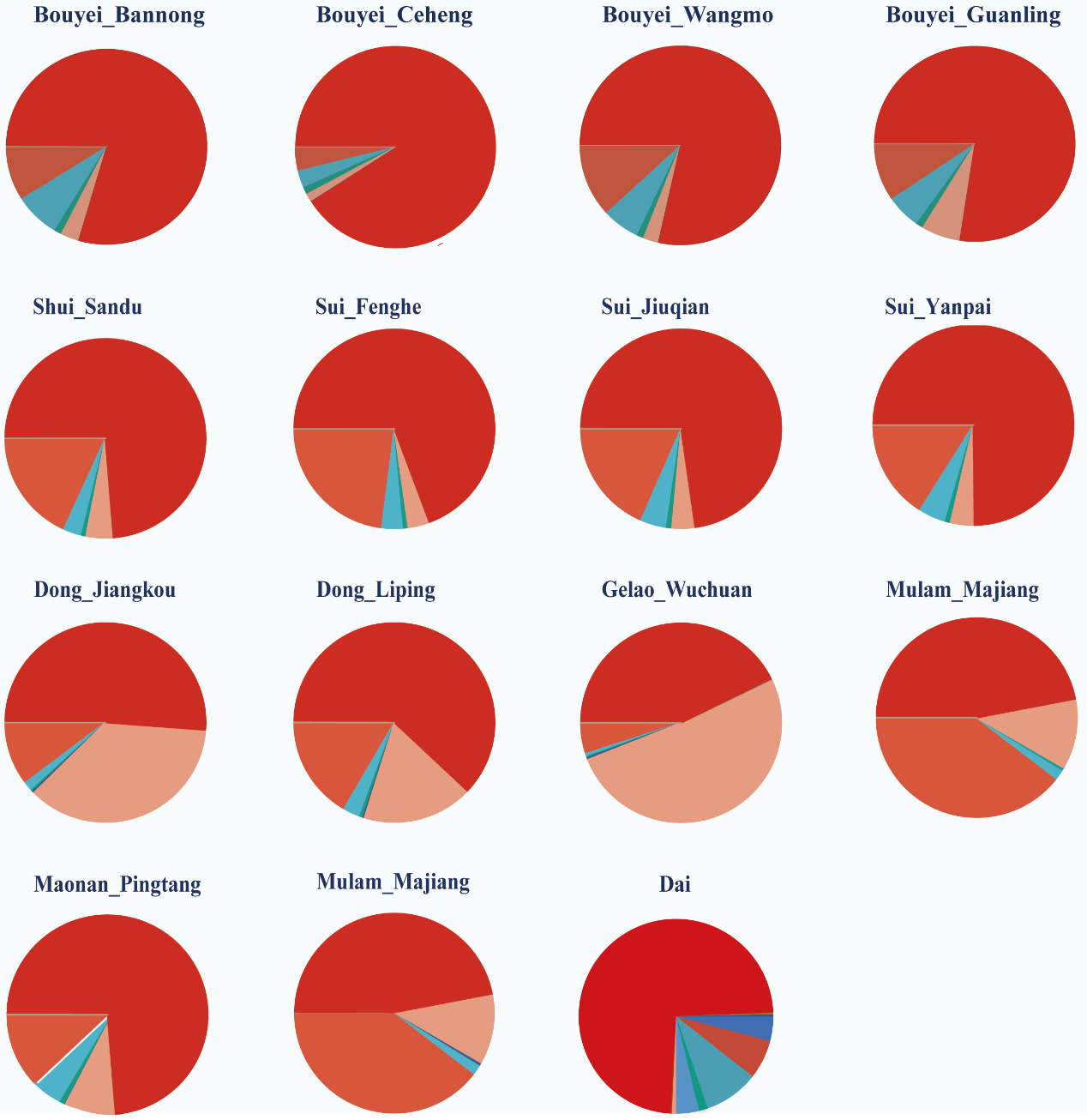


**Figure S2.** **The ancestry composition among fourteen TK populations in Guizhou.**

Results of the proportions of genetic components for 14 TK populations in Guizhou based on the high-density merged WGS dataset.


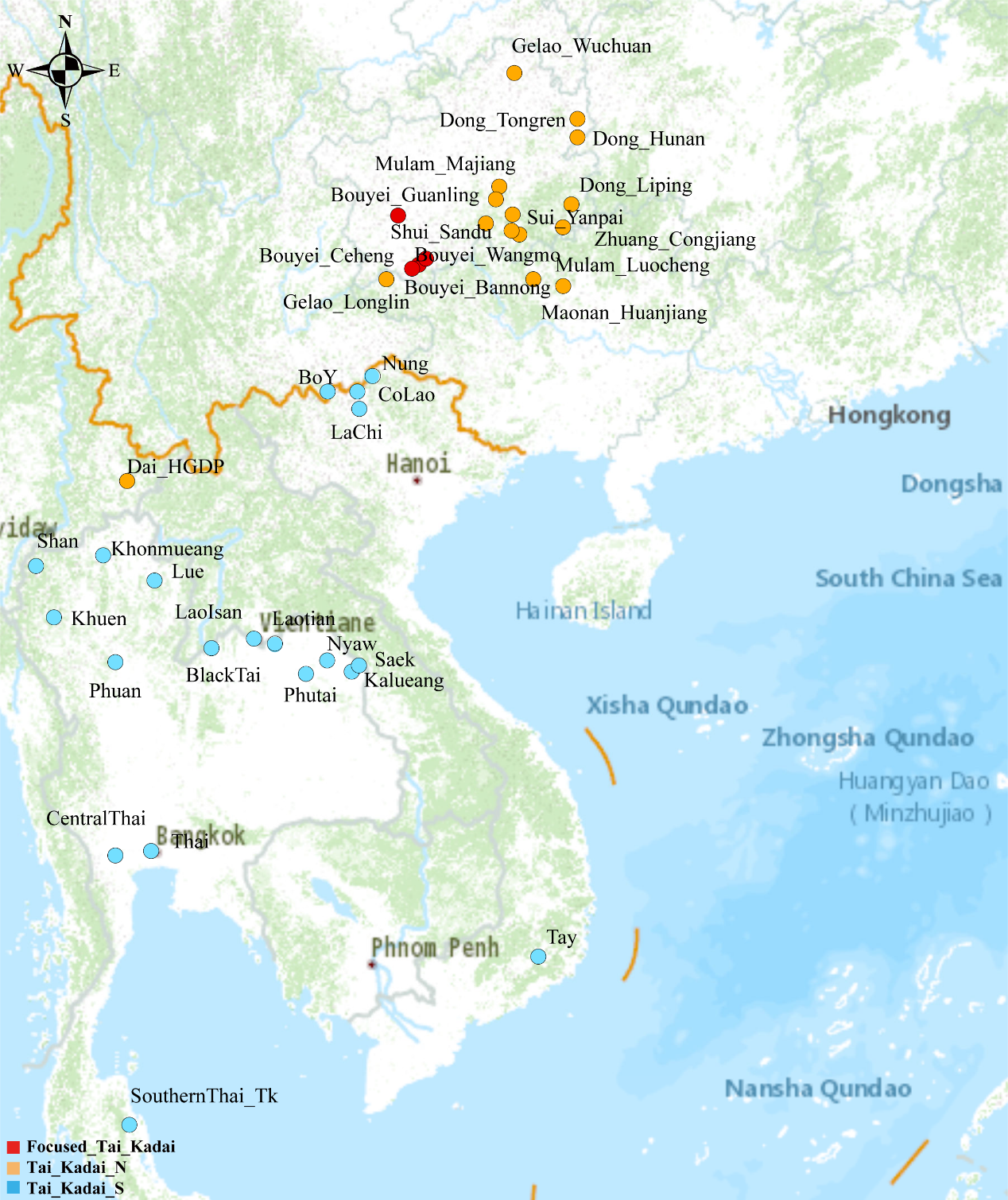


**Figure S3. Sample location of the 39 TK populations.**

The geographic locations of 39 TK populations in the current study include Southern China and SEA based on the low-density merged HO dataset. Red circles represented four Bouyei populations; orange circles represented TK populations in China; blue circles represented other populations from SEA.


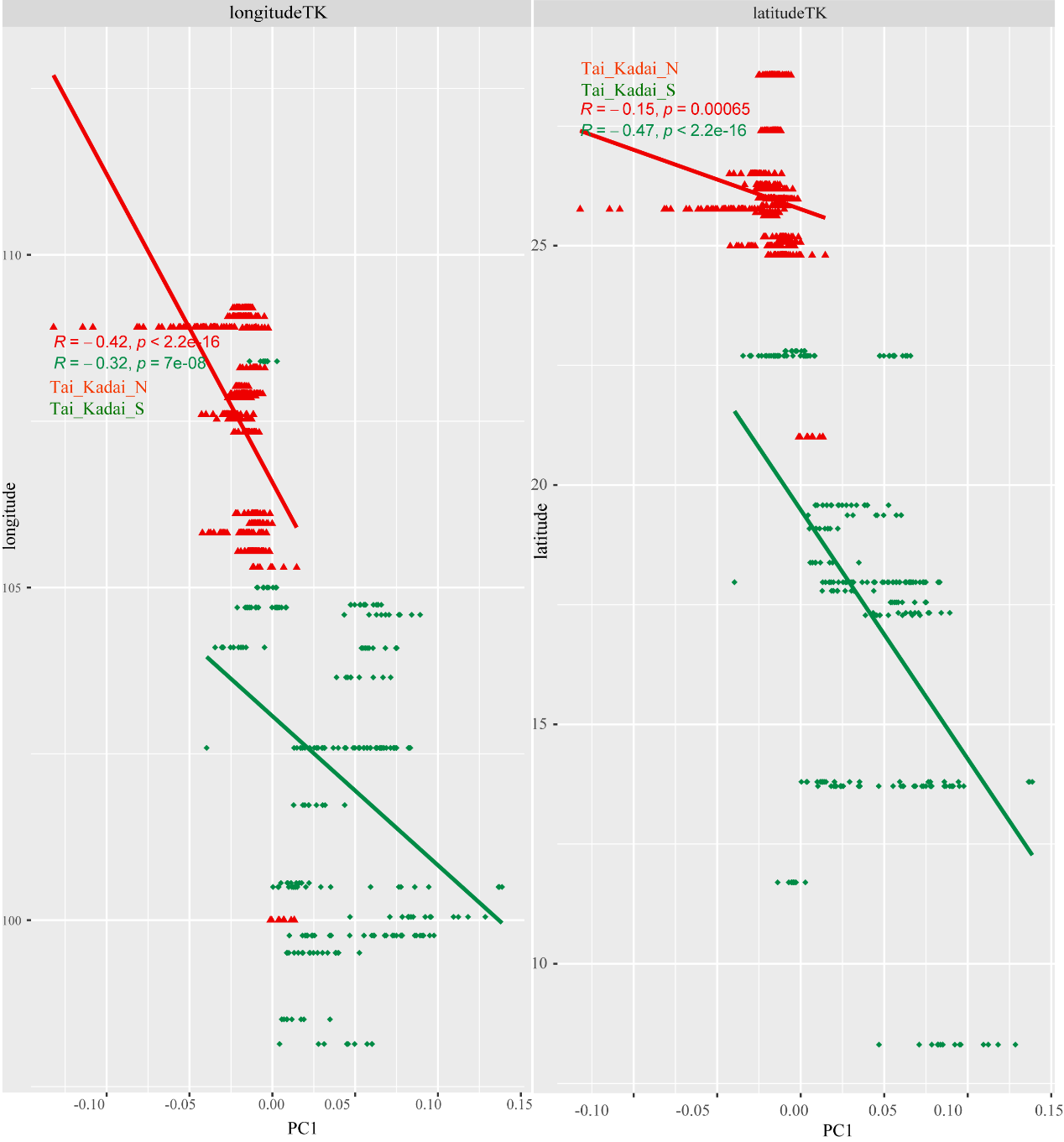


**Figure S4. The genetic substructure within 39 TK populations.**

Person's correlation analysis between geographical coordinates (latitude and longitude)and the values of PC1 among 39 TK populations.


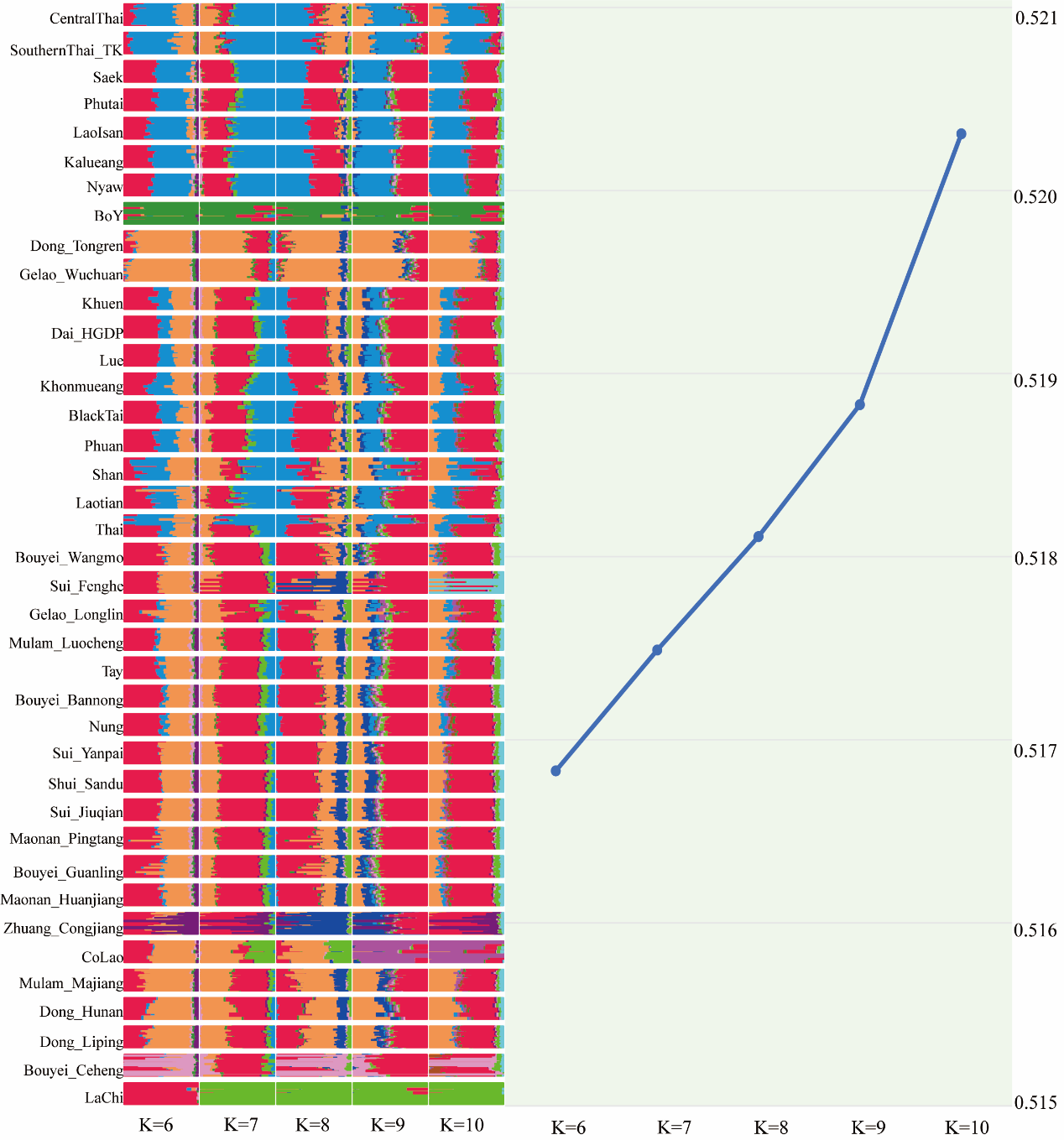


**Figure S5. Model-based ADMIXTURE analysis of the 39 TK populations with the 6–10 ancestral sources.**

When K=6, the cross-value was least. Each increase in the value of K between 6 and 10 resulted in a single population being distinguished. The right line graph shows the trend of the cross-error values between 6 and 10.


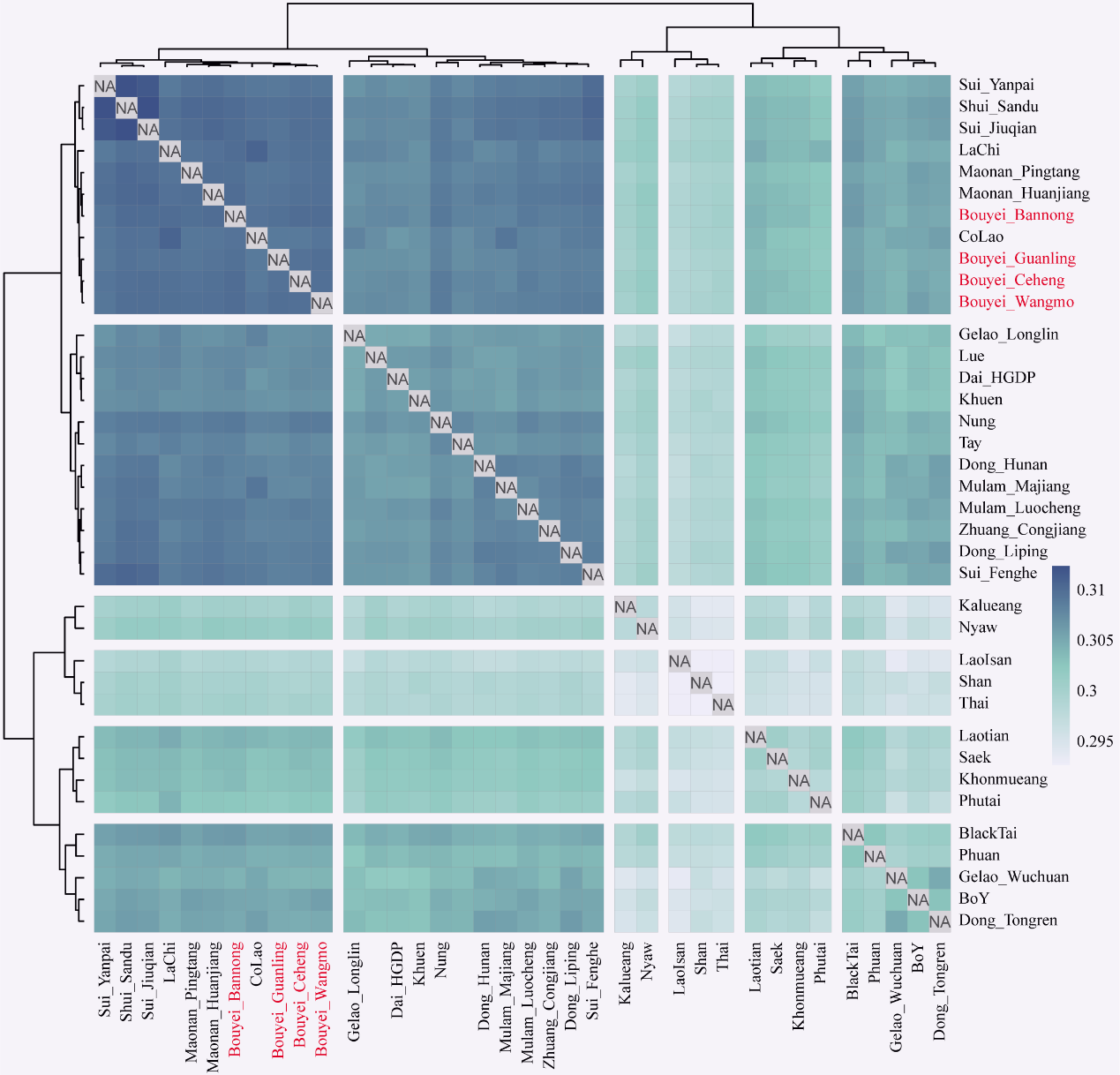


**Figure S6. Genetic affinities of the Bouyei populations based on the** **Outgroup-*f*_3_ and shared genetic drift.**

Heatmap of the shared genetic drift inferred from outgroup *f*_3_-statistics in the form *f*_3_(Studied Bouyei, TK; Mbuti) among 37 populations from Southern China and SEA except for CentralThai and SouthernThai_TK.


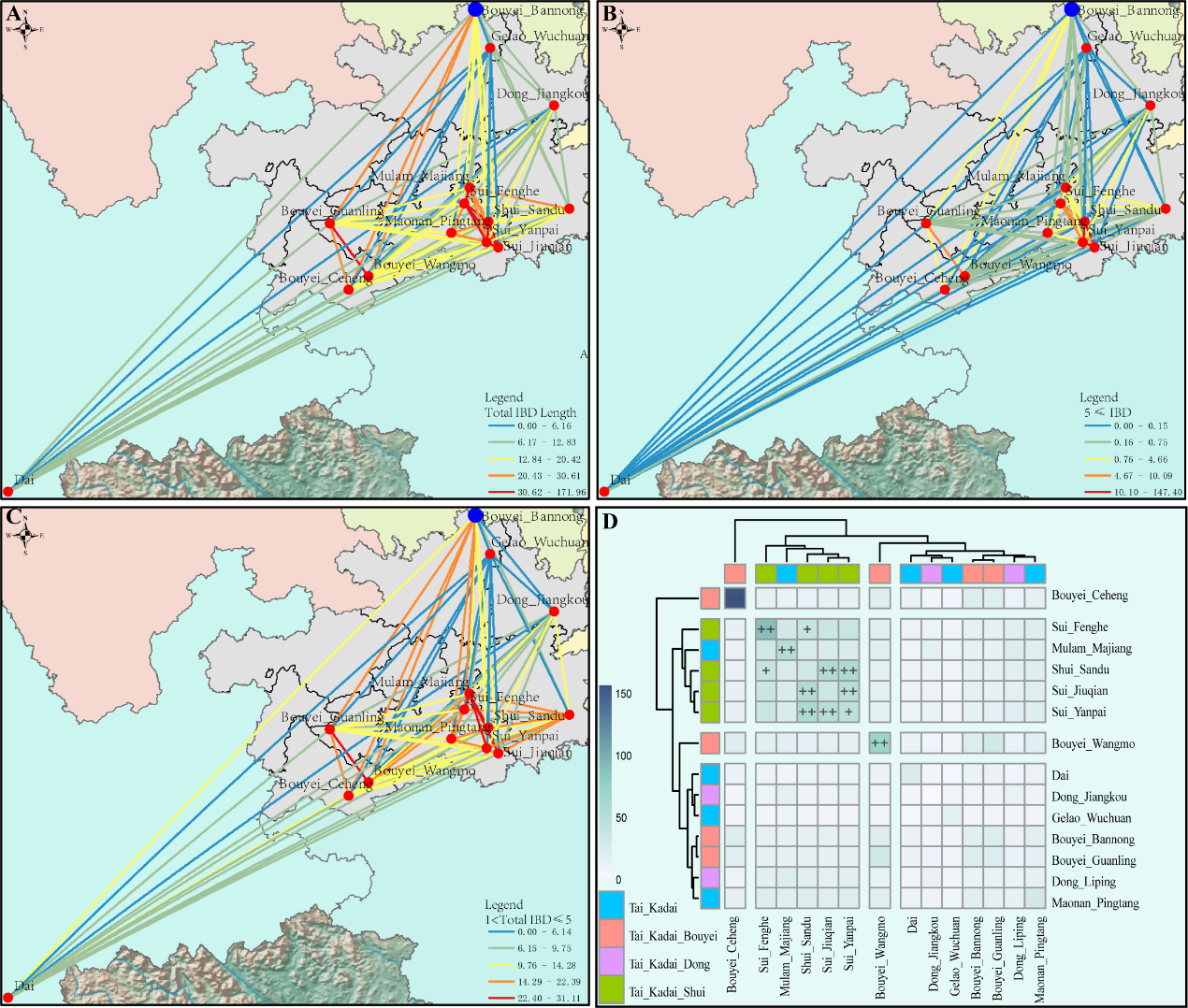


**Figure S7.** **Pairwise identity-by-descent (IBD) networks within the 14 populations in Guizhou.**

Shared identity by descent (IBD) fragments in different length ranges of Bouyei and other TK populations. Different colors represented the different total number of shared IBD fragments between populations. (**A-C**) (**A**)The total IBD length in 14 TK populations in Guizhou; (**B**) The pairwise IBD sharing average length between 14 populations more than 5; (**C**) The pairwise IBD sharing average length between 14 populations more than 1 but less than 5; (**D**) The pairwise IBD sharing average length between 14 populations all.


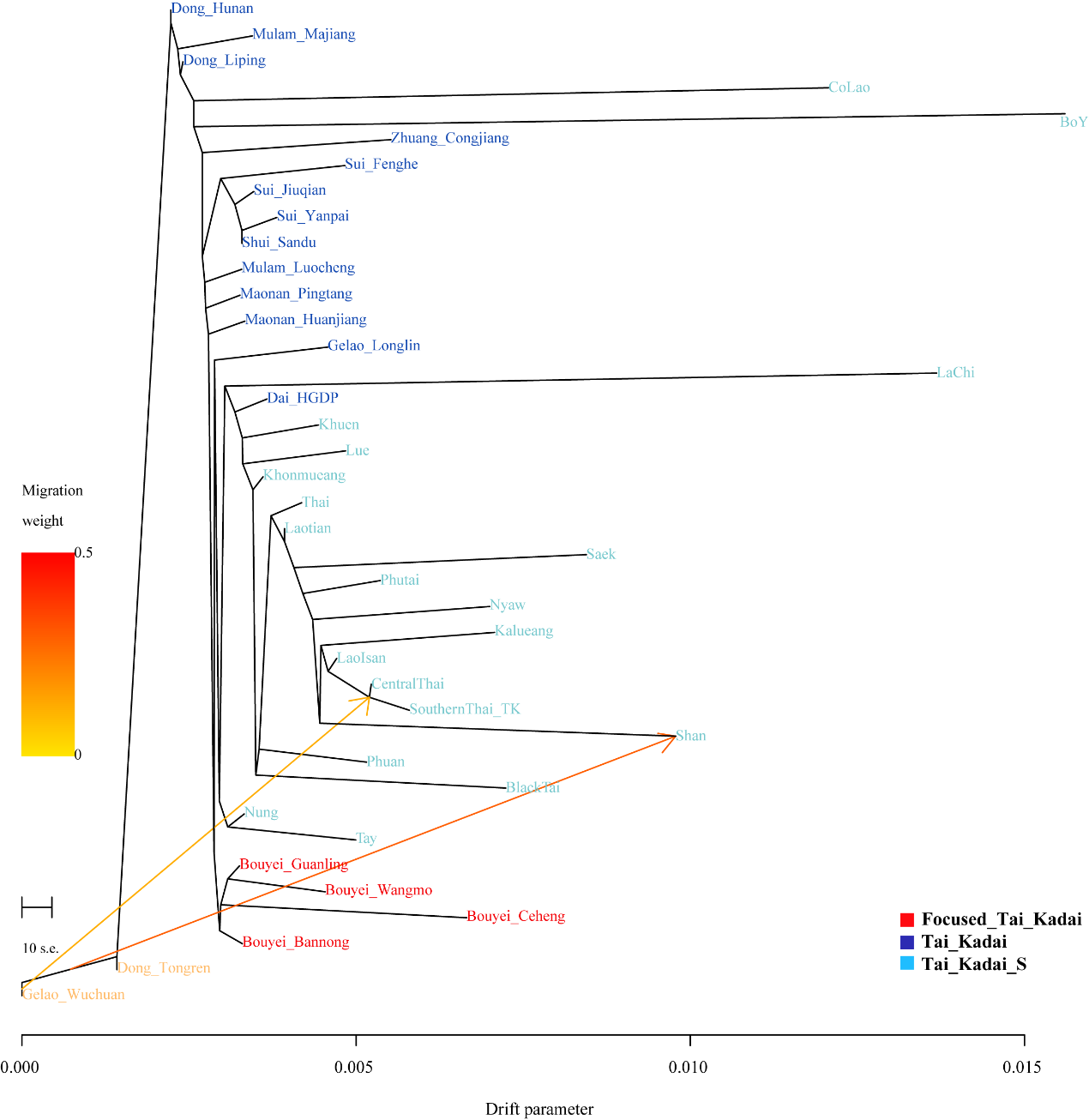


**Figure S8.** **The phylogenetic tree within 39 TK populations.**

The phylogenetic relationship showed the close genetic affinity between Bouyei and other TK-speaking populations.


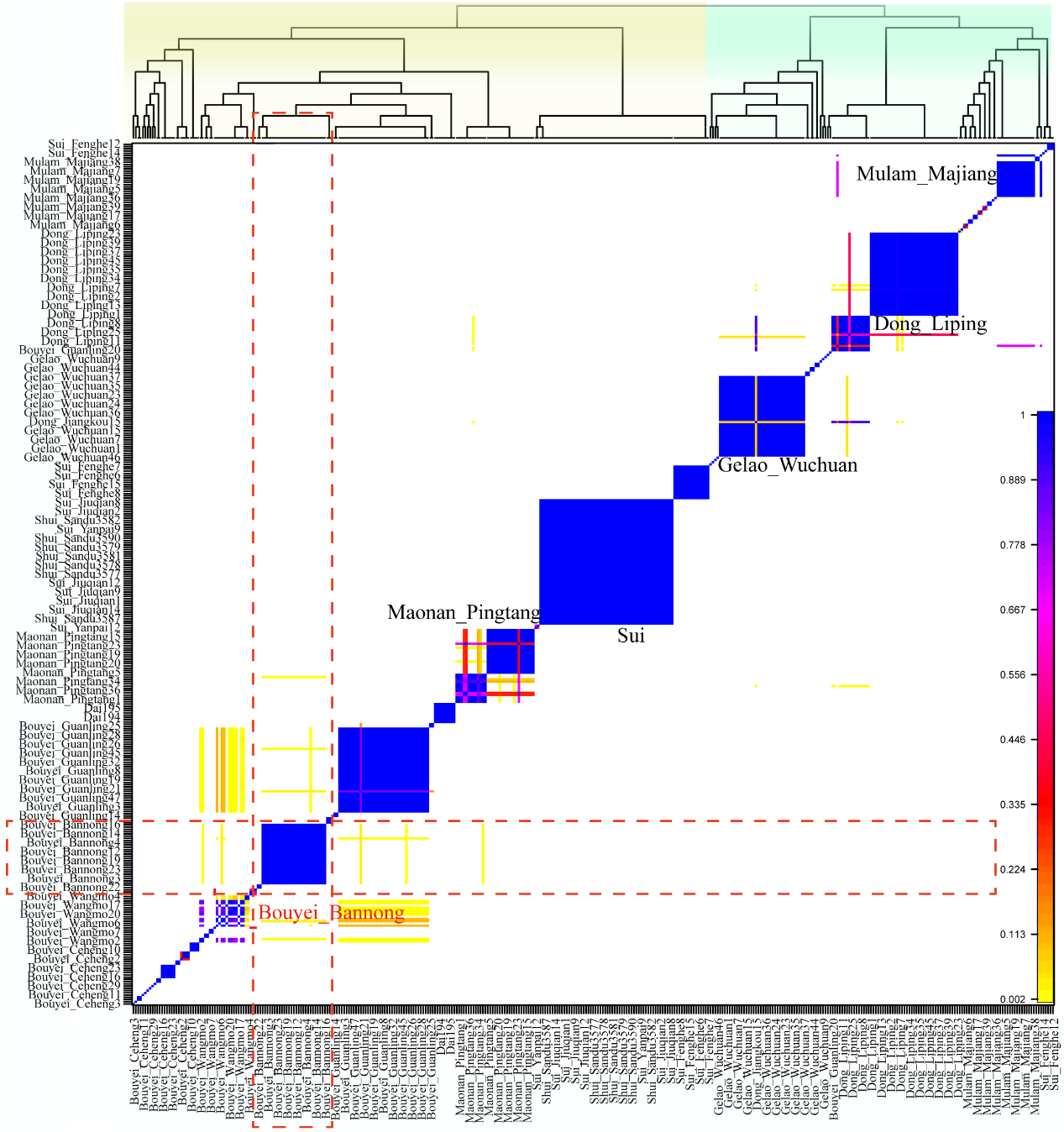


**Figure S9: Clustering patterns of TK individuals in Guizhou based on the pa****irwise coincidence matrix.**

Pairwise coincidence matrix at individual level inferred by fineSTRUCTURE with extremely high (dark blue) and low (yellow) values. In general, it can be divided into two clusters from FullDendrogram, and target groups (Bouyei_Bannong) were marked in red.


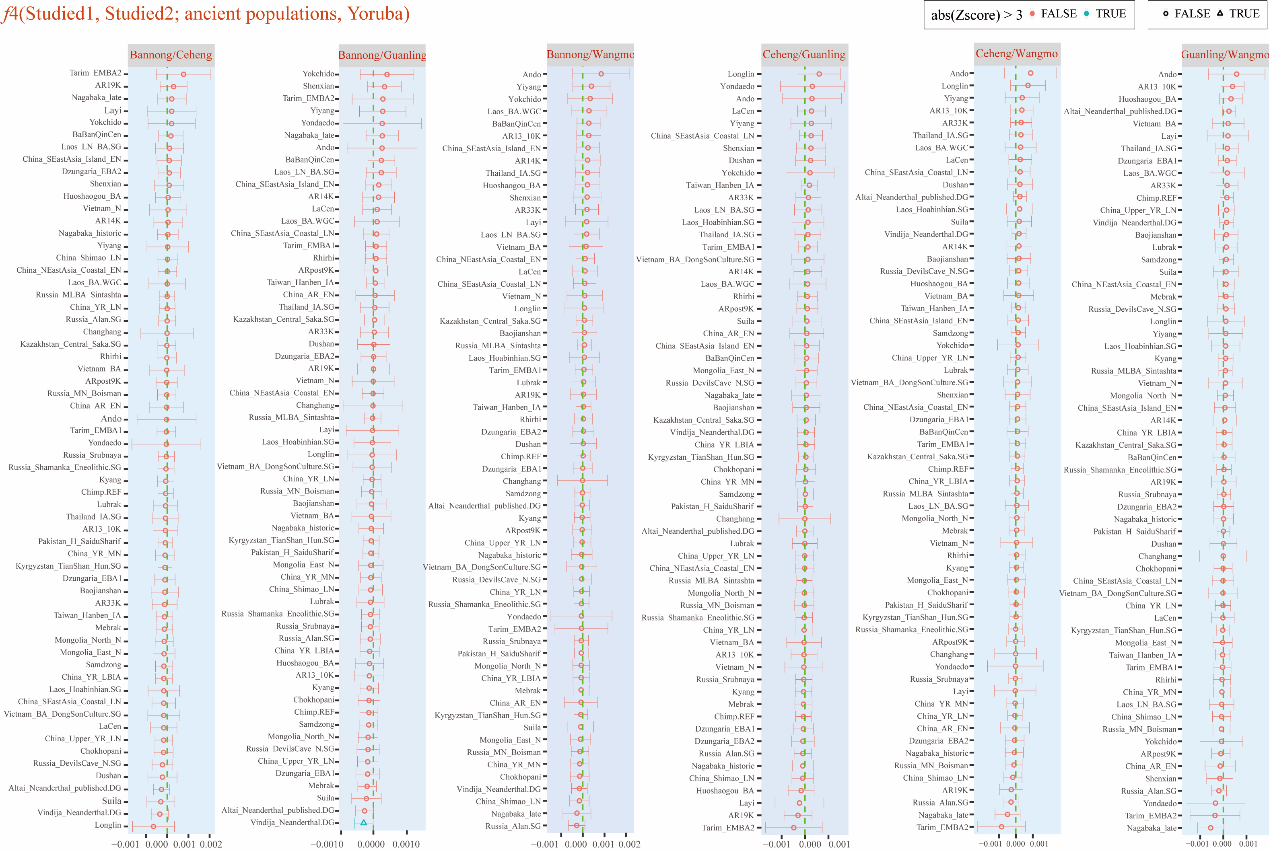


**Figure S10: *F*_4_-statistic test for** **homogeneity and heterogeneity in four Bouyei groups.**

*F*_4_-statistics in the form of *f*_4_(Studied1, Studied2; ancient populations, Mbuti). The |Z |values of *f*_4_ under 3 standard errors indicated four Bouyei groups are genetic homogeneity. Different pairwise population pairs were used to explore homogeneity and heterogeneity in four Bouyei groups.


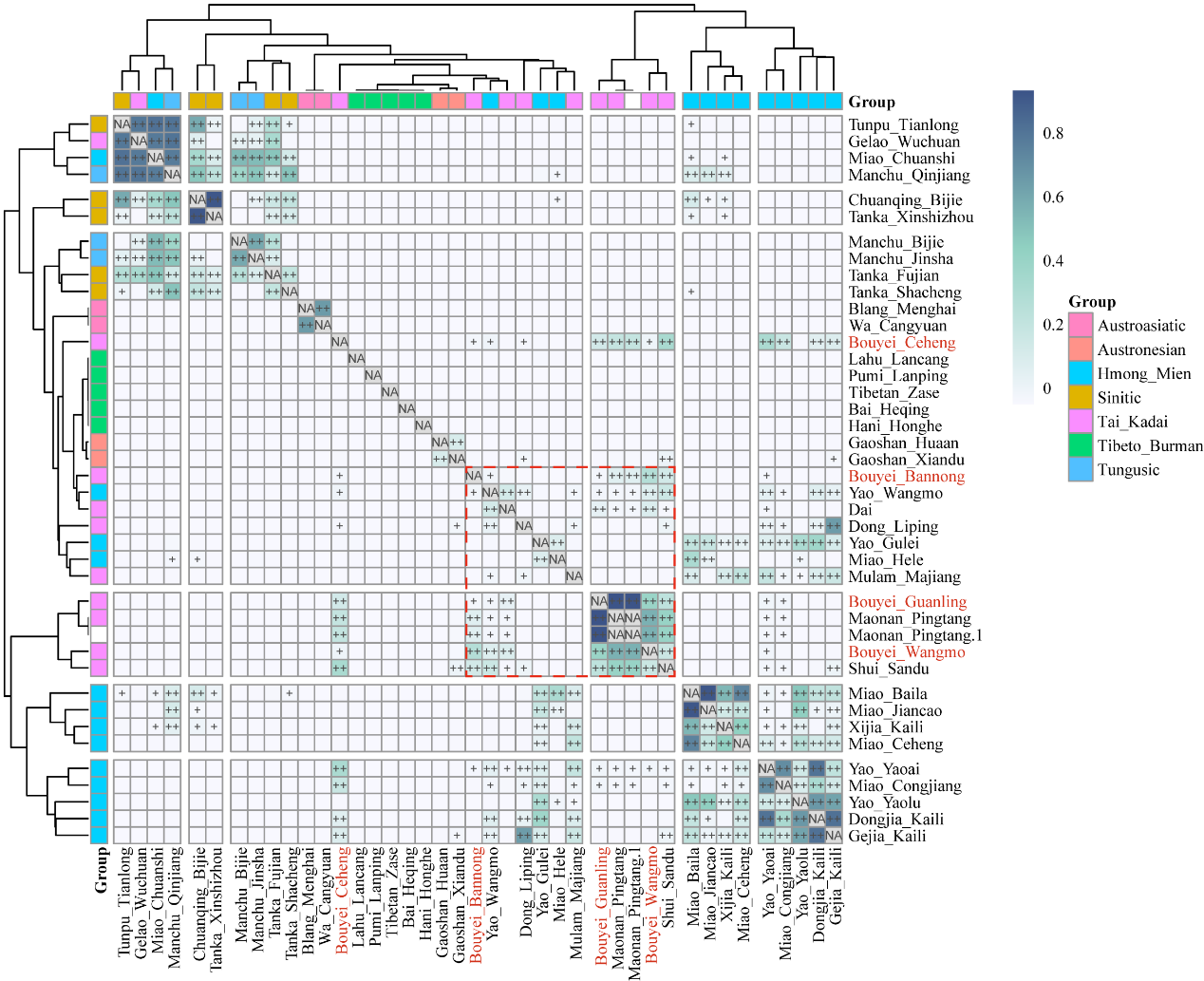


**Figure S11: Pairwise qpWave test for homogeneity and heterogeneity between Bouyei and other East Asian populations.**

Pairwise qpWave results showed the genetic homogeneity and heterogeneity between Meta-Bouyei and other modern populations. The label of “+” denoted p values larger than 0.01.


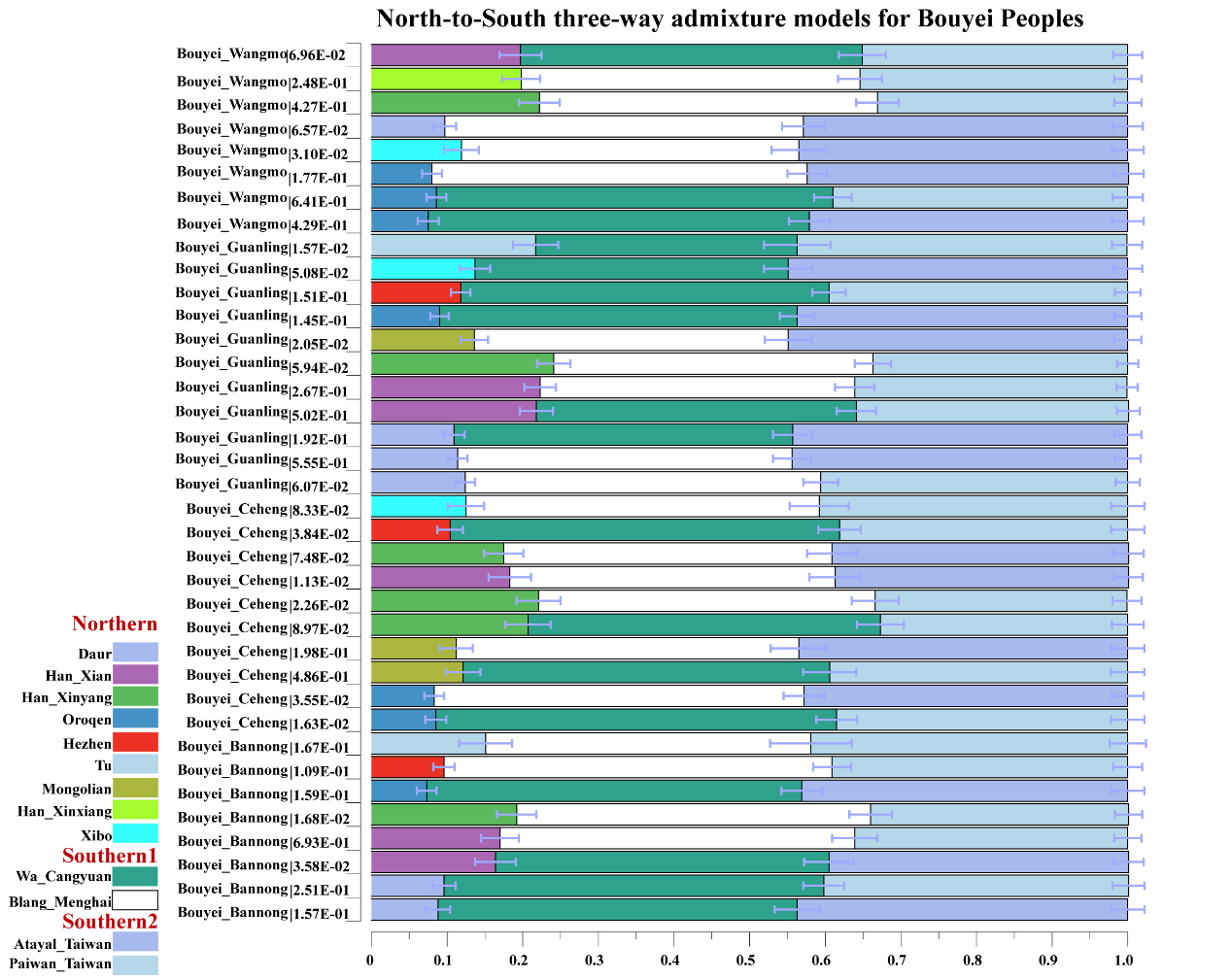


**Figure S12: Admixture proportion of ancestry groups from modern inferred using qpAdm.**

Three-way admixture models showed that modern Northern and Southern populations contributed to the formation of four Bouyei people. The error bar indicated the standard errors of predicted proportions of ancestors obtained from qpAdm.


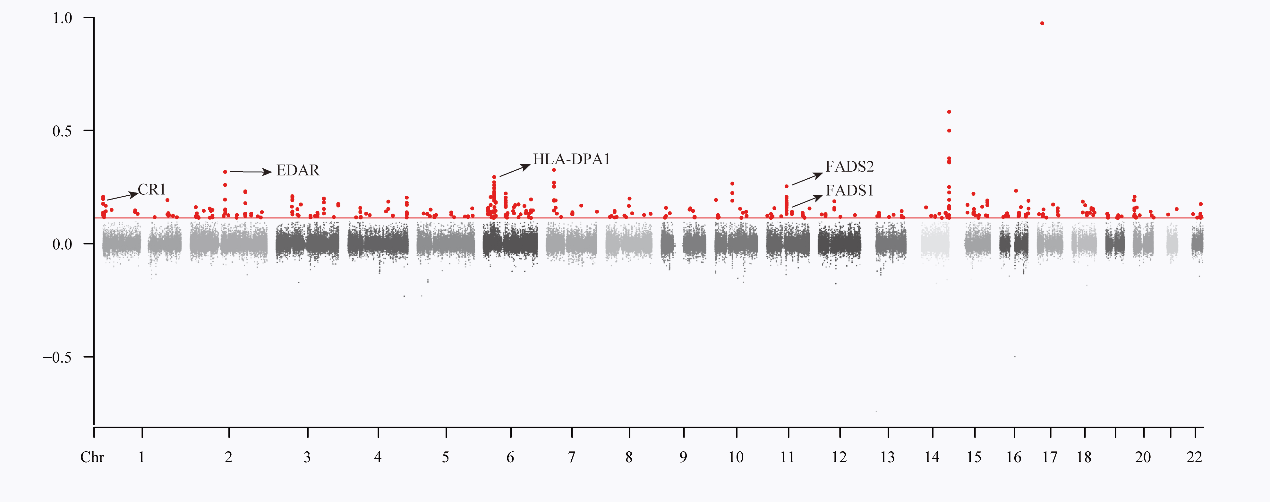


**Figure S13: Positive natural selection signals.**

Manhattan plot showing the PBS values in genome-wide scanned for Bouyei population in Guizhou, using the Shaanxi_Han and European as ingroup and outgroup reference populations. The 99.9th percentiles of the PBS distribution were shown as red lines. PBS values over the 99.9th percentile were marked in red, and PBS values under the 99.9th percentile were colored as dark dots. Otherwise, some of the genes were labeled with its name.


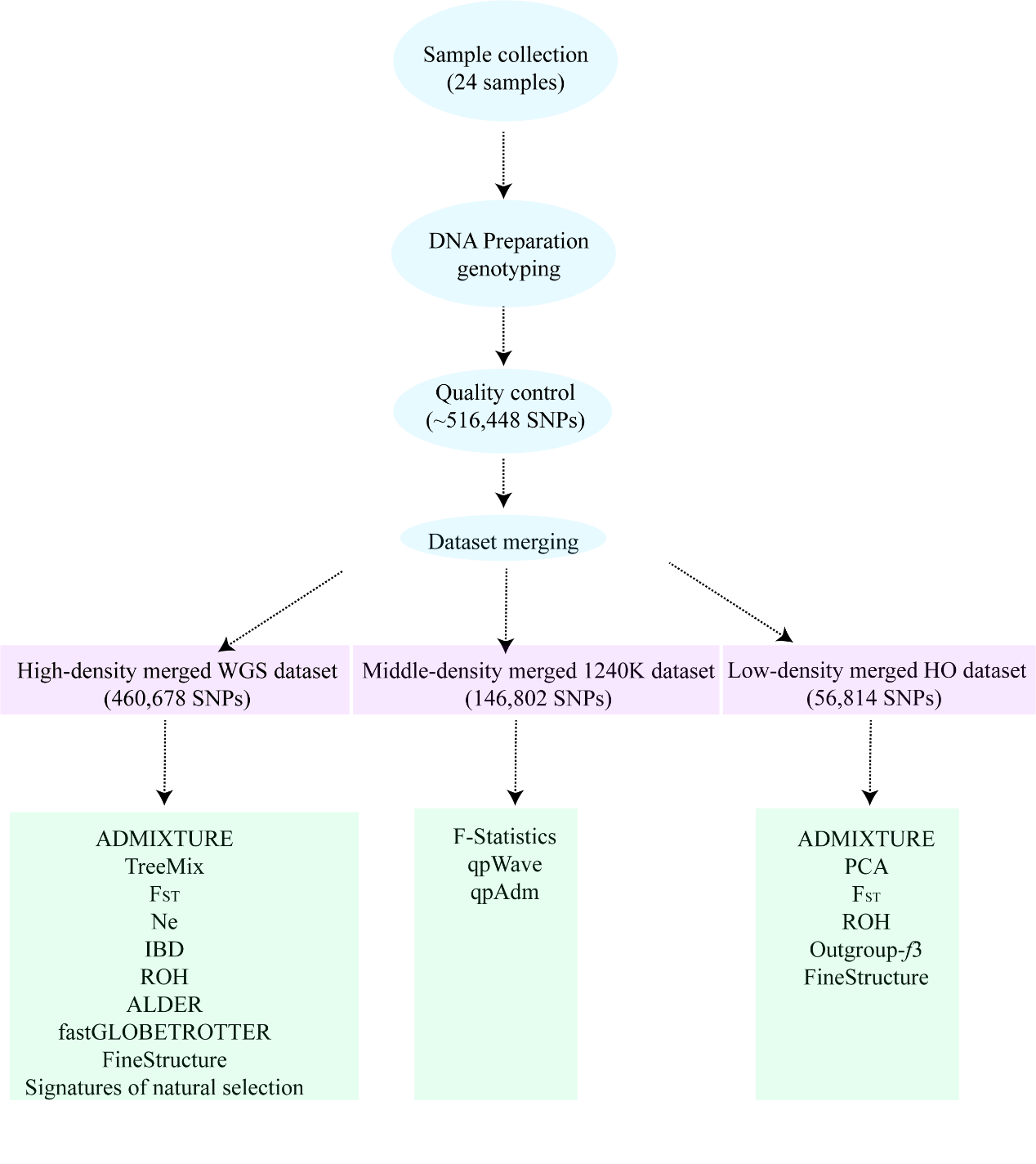


**Figure S14:** **Process analysis diagram.**

The figure above shows the flow of our analysis, including sample collection, quality control, merging datasets, and the analyzes used, which are all mentioned in the method.
